# The effects of point defect type, location, and density on the Schottky barrier height of Au/MoS_2_ heterojunction: a first-principles study

**DOI:** 10.1038/s41598-022-22913-7

**Published:** 2022-10-26

**Authors:** Viacheslav Sorkin, Hangbo Zhou, Zhi Gen Yu, Kah-Wee Ang, Yong-Wei Zhang

**Affiliations:** 1 Institute of High-Performance Computing, A*STAR, 1 Fusionopolis Way, Singapore, 138632 Singapore; 2 Department of Electrical and Computer Engineering, National University of Singapore, 4 Engineering Drive 3, Singapore, 117583 Singapore; 3 Institute of Materials Research and Engineering, A*STAR, 2 Fusionopolis Way, Singapore, 138634 Singapore

**Keywords:** Applied physics, Physics, Electronics, photonics and device physics, Electronic and spintronic devices

## Abstract

Using DFT calculations, we investigate the effects of the type, location, and density of point defects in monolayer MoS_2_ on electronic structures and Schottky barrier heights (SBH) of Au/MoS_2_ heterojunction. Three types of point defects in monolayer MoS_2_, that is, S monovacancy, S divacancy and Mo_S_ (Mo substitution at S site) antisite defects, are considered. The following findings are revealed: (1) The SBH for the monolayer MoS_2_ with these defects is universally higher than that for its defect-free counterpart. (2) S divacancy and Mo_S_ antisite defects increase the SBH to a larger extent than S monovacancy. (3) A defect located in the inner sublayer of MoS_2_, which is adjacent to Au substrate, increases the SBH to a larger extent than that in the outer sublayer of MoS_2_. (4) An increase in defect density increases the SBH. These findings indicate a large variation of SBH with the defect type, location, and concentration. We also compare our results with previously experimentally measured SBH for Au/MoS_2_ contact and postulate possible reasons for the large differences among existing experimental measurements and between experimental measurements and theoretical predictions. The findings and insights revealed here may provide practical guidelines for modulation and optimization of SBH in Au/MoS_2_ and similar heterojunctions via defect engineering.

## Introduction

Metal–semiconductor junctions have been widely used in modern electronic devices. In such a junction, a Schottky barrier, which is a potential energy barrier for electron or hole, can be formed. The Schottky barrier height (SBH) is essential in rectifying electrical current characteristics^1^. Recently, a new type of computing devices based on artificial synapses (e.g., memtransistors, resistive synaptic switches, memristors, etc.) that mimic the biological neural systems have attracted significant research interests^2^. Of particular interest is the exploration of semiconducting two-dimensional (2D) materials for such artificial synapses, and molybdenum disulfide (MoS_2_) monolayer, which is a typic semiconducting 2D material, is often used, and its junction with a metallic electrode becomes a principal building block^3–6^. Since SBH plays an important role in modulating charge carrier transport, switching characteristics^7^ and device performance^8–10^, accurately setting and adjusting the SBH is of critical importance for the control of charge transport in MoS_2_ and the design of memory switching in MoS_2_-based devices.

Yet, accurate control of SBH is still a challenge in designing semiconductor-based high-performance nanoscale electronics. It is known that many factors can affect the SBH of metal/MoS_2_ junctions, such as strong Fermi-level pinning (FLP)^8,11–13^, electronic band alignment^14,15^ and dipole formation due to the charge redistribution at the contact^11,16,17^, bond formation between MoS_2_ and the underlying substrate^18^, push-back effect^10,11^, dielectric screening due to MoS_2_ layer^15^, quantum confinement (the out-of-plane interactions between MoS_2_ monolayer and metallic surface can strongly modify the boundary condition for quantum confinement on one side of the MoS_2_)^19^, interfacial stress and strain^20,21^ and the presence of defects in MoS_2_ layer and metallic substrate (e.g., point and line defects of various types at different concentrations and spatial distributions)^22–26^. Due to the complexity, there is a large scattering in existing experimental measurements of SBH, and there is a large discrepancy between the experimental measurements and the existing theoretical predictions^13–16^. The reconciliation between these discrepancies so far has not been achieved.

Two experimental techniques are widely used to prepare MoS_2_ samples: CVD growth^27^ and mechanical exfoliation^28,29^. Compared with the mechanical exfoliation, the CVD growth, occurring at relatively high temperatures, induces various native defects, including point defects, grain boundaries and edges^30^. The equilibrium concentration of point defects is determined by their formation energies and growth conditions (temperature, pressure, and chemical potential). Hence, the experimentally observed defect densities vary strongly from experiment to experiment^26^. The estimated sulfur vacancy density^31–37^ is in the range ${n}_{v}$~10^8^–10^11^ cm^−2^. The MoS_2_ monolayers with various defects are used in metal/MoS_2_ heterojunctions, and the experimentally measured SBH values often fall in a broad range. For example, the SBH for Au/MoS_2_ contact varies between 0.06 eV and 0.92 eV^1,8,38,39^. It is possible that the defective and inhomogeneous MoS_2_ samples used in experiments can be the reason for the observed scattering of the SBH values and the deviations from the intrinsic value of defect-free MoS_2_/metal contact^36,40,41^.

Moreover, it is common to apply the electrode deposition to create the metal/MoS_2_ junction, in which the deposited ‘‘high energy’’ metal atoms can damage the crystal lattice of MoS_2_. This deposition can lead to a substantial chemical disorder, namely formation of numerous S and Mo vacancies, and metallic-like defects (metallic impurities) at the interface^3,15,42^. The chemical disorder can have a profound effect on both the SBH and FLP. In contrast, when atomically flat metal thin films are laminated onto MoS_2_ monolayer (without direct chemical bonding) by using the damage-free electrode transfer technique^1^, the observed interface is effectively free from chemical disorder and FLP.

The effect of the point defects on the SBH was studied by using DFT, primarily focusing on the effect of vacancies^5,11,38,39,43–48^. Feng et al.^12^ studied the Ti/MoS_2_ heterojunctions with S and Mo vacancies in MoS_2_. They found that S vacancies reduce the SBH, while Mo vacancies completely remove the Schottky barrier. The effect is due to the strong interactions of the vacancies with the underlying substrate. Yun and Lee^48^ examined SBH tuning at Co/MoS_2_ and Ni/MoS_2_ heterojunctions through S and Mo vacancies. It was found that the SBH significantly increases (by ~ 30%) due to Mo vacancies as compared to the defect-free cases; and only slightly decreases (by ~ 5%) due to S vacancies. Mo-vacancies are favorable for the p-type contact, whereas S-vacancies for the n-type. Yang et al.^23^ found that at relatively low concentrations of S vacancies, the MoS_2_ monolayer is an electron acceptor in the Au/MoS_2_ contact, while at higher concentrations an electron donor.

Qui et al.^43^ investigated the effect of S and Mo monovacancies on the SBH at the Au/MoS_2_ heterojunction and found that there is a minor increase (by ~ 5%) in the SBH due to S vacancies at defect concentration ${n}_{{V}_{s}}\sim 2\mathrm{\%}.$ The effect of Mo vacancies is significantly stronger since the Schottky barrier height vanishes at same defect concentration. They found that chemical bonds are formed between the monolayer and its underlying substrate, resulting in a transformation of Au/MoS_2_ junction from a Schottky contact to an Ohmic contact^44^. Su et al.^44^ confirmed that the SBH can be eliminated by Mo vacancies at a critical concentration, while S vacancies increase the SBH at Pt/MoS_2_ heterojunction^45^. Fang et al.^46^ also found that the SBH increased in MoS_2_ when contacted with Mg, Al, In, and Au, while reduced in defective MoS_2_ when contacted with Cu, Ag, and Pd.

Experimental studies^19,41^ demonstrated that MoS_2_ samples may contain different types of defects, for example, S monovacancy, S divacancy and antisite defect (in which an S atom is substituted by a Mo atom or vice versa). For the same type of defect, it may be found at different locations within MoS_2_ monolayer, for example, at the top or the bottom sublayer. Also, the defect concentration is subject to variation. An interesting question is: how do defect type, location, and density in the MoS_2_ layer affect the SBH of a metal/MoS_2_ heterojunction? To answer this question, we choose the Au(111)/MoS_2_ heterojunction to systematically examine these effects by leveraging our expertise in first-principles calculations.

The Au(111) substrate is chosen because of its well-known chemical inertness, strong electronegativity, stability, and affinity to sulfur^23^. Moreover, Au(111) is selected to minimize the effect of lattice mismatch between MoS_2_ monolayer and the underlying Au substrate in the Au/MoS_2_ heterojunction. Both MoS_2_ and Au(111) surfaces have the same lattice structure: the outer surface of MoS_2_ monolayer is a hexagonal lattice of S atoms while the close-packed Au(111) surface is a hexagonal lattice of Au atoms. The mismatch between the two hexagonal lattices is relatively small (~ 6%), hence a comparatively small deformation of Au(111) substrate is sufficient to eliminate the lattice mismatch and form a coherent Au/MoS_2_ heterostructure. For these reasons, Au(111) has been the surface of choice for most Au/MoS_2_ heterojunctions, which have been widely used in experiments and practical applications^22,49,50^.

Three types of point defects are considered: S monovacancies, S divacancies, and Mo_S_ antisite defects. In addition, the same defects located on the outer or inner sublayer of MoS_2_ are also studied and compared. Finally, the effect of defect density per unit area on the SBH is also examined. Ultimately, we would like to find out whether these factors can explain the broad variation in the experimentally measured SBH values and propose possible strategies to control the SBH.

Two different first-principles-based methods can be used to calculate the SBH in the MoS_2_/MoS_2_ contact. The first method is based on the projection of electronic band structure of MoS_2_ layer taken from the Au/MoS_2_ heterojunction on the band structure of the entire junction. The second method is based on the Schottky–Mott (SM) rule^15^, which requires to calculate the work function (${W}_{Au}$) for Au substrate, electronic affinity energy (EAE) for MoS_2_ monolayer, and the step in electrostatic (Hartree) potential of Au/MoS_2_ heterojunction. Since the results obtained by these methods can differ from each other, in this study, we employ both methods, compare their predictions, and assess their reliability as well as accuracy.

Our first-principles calculations show that both the calculation methods predict the same trend for the SBH, and with proper treatments, the two methods can predict nearly the same results. We also show that the SBH of the Au/MoS_2_ contact is affected by defect type, location, and density in MoS_2_ monolayer. More specifically, the SBH in the Au/MoS_2_ contact with the defective MoS_2_ monolayers is universally higher than that of the defect-free monolayer. Among the defects considered, Mo_S_ antisite defect and S divacancy significantly increase the SBH, while it is only weakly affected by S monovacancy. Moreover, the defects in the inner sublayer have more influence on SBH than those in the outer sublayer. Finally, an increase in the defect density noticeably increases the SBH. Our study suggests that the reported variations in the experimentally measured SBH for Au/MoS_2_ contact can be accounted (to a certain degree) by the variations in the type, location, and density of point defects in MoS_2_ monolayer. However, the predicted SBH values are ubiquitously higher than the experimentally measured values. We suggest that the lower SBH values observed in experiments may be due to the difference in experimental samples. The present study indicates that the value of SBH can be altered via defect engineering in the MoS_2_ layer. Our findings provide a guide for tuning the SBH in the Au/MoS_2_ heterojunctions.

## Computational model

Different types of point (single and double vacancies, antisite defects, etc.) and topological (Stone–Wales) defects can be created in the MoS2 monolayer at the Au/MoS_2_ heterojunction (see Table 1). Here, we examined only three types of point defects with low formation energy, which can be experimentally observed with a high probability. The effect of other defects with higher formation energy, such as Mo vacancy, V_MoS2_ and V_MoS3_ cluster vacancy and Stone–Wales defects are thus not considered. These three types of point defects are S monovacancy, S divacancy, and Mo_S_ antisite defect (see Table 1).

**Table 1:** Various types of defects in MoS_2_ monolayer and their formation energies obtained by DFT calculations.

Defect type	Notation	Formation energy (eV)	References
Single S vacancy	V_S_	1.5–2.8	^22,24,47^
Double S vacancy	DV_S_	3.2–5.4	^22,30,51^
Antisite defect	Mo_S_	4.2–6.2	^47^
Single Mo vacancy	V_Mo_	4.9–8.2	^30,47^
SW defect	SW	5.9–6.6	^52^
Cluster vacancy type I	V_MoS2_	7.7	^30^
Cluster vacancy type II	V_MoS3_	8.2	^30^

For our DFT calculations, we selected three different types of point defects with a relatively low formation energy: (1) S monovacancy with formation energy of ${E}_{S}^{vac}$ = 1.55 eV^22,47^ in Mo-rich limit (deficit of S-atoms) and ${E}_{S}^{vac}$ = 2.81 eV^22,24,47^ in S-rich limit (deficit of Mo-atoms), (2) S divacancy with ${E}_{2S}^{vac}$ = 3.2 eV^30,51^ in Mo-rich limit and ${E}_{2S}^{vac}$ = 5.44 eV in S-rich limit^22^. Since the formation energy of an S divacancy is roughly twice of S monovacancy, the monovacancies in MoS_2_ in contrast to graphene do not have a strong tendency to merge into divacancies^30^. (3) Mo_S_ antisite defect with ${E}_{Mo\to S}^{sub}$ = 4.2 eV^47^ in S-rich limit and ${E}_{Mo\to S}^{sub}$ = 6.2 eV^47^ in Mo-rich limit. We note that when MoS_2_ monolayer interacts with Au substrate, the formation energies of these point defects are slightly higher (for example, the formation energy of S monovacancy increases by ~ 7%^22^).

Since the formation energy of a Mo monovacancy is ${E}_{Mo}^{vac}$ = 8.2 eV in Mo-rich limit^47^ and ${E}_{Mo}^{vac}$ = 4.9 eV in S-rich limit^30^, once a Mo monovacancy is formed, there is a strong tendency to form S vacancies from its neighbouring S atoms (since the formation energy of S vacancy around a Mo vacancy is only ${E}_{S}^{vac}$ = 1.1 eV^30^). Therefore, Mo monovacancies are not observed experimentally alone, but as clusters of vacancies which appear via merging of S and Mo monovacancies, such as: V_MoS2_ with ${E}_{Mo{S}_{2}}^{vac}$ = 8.2 eV and V_MoS3_ with ${E}_{Mo{S}_{3}}^{vac}$ = 7.7 eV in S-rich limit^30^. Yet, the clusters of vacancies are unstable^22^, especially when MoS_2_ layer is supported by Au substrate. For this reason, S monovacancies are frequently observed experimentally, but Mo monovacancies are only occasionally found^30^.

**Figure 1:**
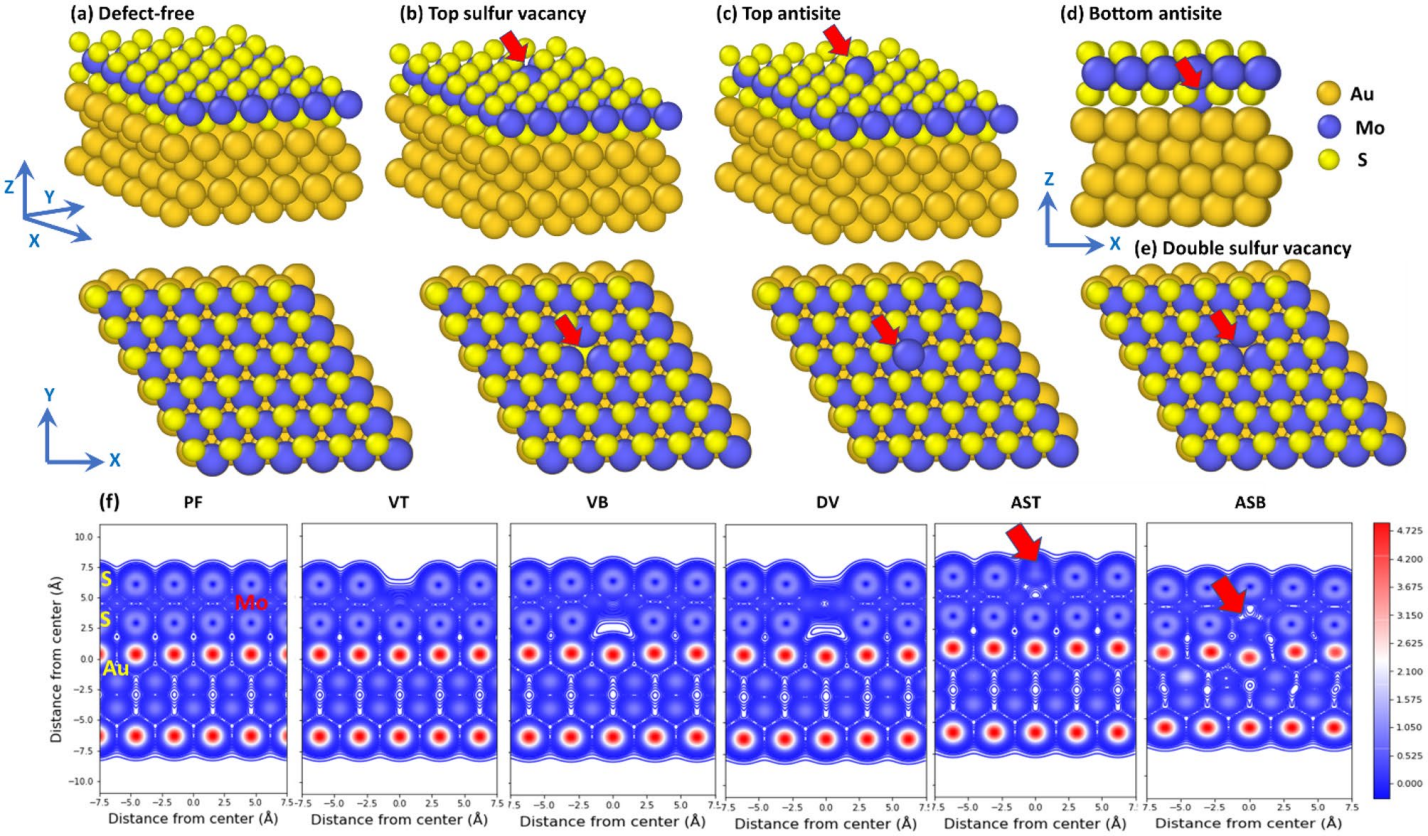
The side and top views of the Au(111)/MoS_2_ 6 × 6x4 samples: (**a**) defect-free sample (PF), (b) sample with an S monovacancy in the top sublayer (VT), (**c**) sample with an antisite defect in the top sublayer (AST), and (**d**) sample with an antisite defect in the bottom sublayer (ASB), and (**e**) sample with a double S vacancy (DV). Mo atoms marked by blue, small yellow spheres correspond to S-atoms, and large ones to Au-atoms. (**f**) The planar charge density distribution around the point defects in the Au/MoS_2_ 5 × 5 × 4 samples (bottom panel): PF, defect-free MoS_2_; VT, MoS_2_ with an S monovacancy in the top sublayer; VB, MoS_2_ with an S monovacancy in the bottom sublayer; DV, MoS_2_ with a double S vacancy; AST, MoS_2_ with an antisite defect at the top sublayer; ASB, MoS_2_ with an antisite defect in the bottom sublayer. Color bar indicates the charge density values. Red arrow indicates the defect position.

As the first step, we constructed and optimized the Au(111)/MoS_2_ samples. The following six Au/MoS_2_ samples were constructed (see Fig. 1): (1) defect-free (PF) MoS_2_, (2) MoS_2_ with a sulfur monovacancy in the top sublayer (VT), (3) MoS_2_ with a sulfur monovacancy in the bottom sublayer (VB), (4) MoS_2_ with a sulfur divacancy (DV), (5) MoS_2_ with a Mo_S_ antisite defect at the top sublayer (AST), and (6) MoS_2_ with a Mo_S_ antisite defect in the bottom sublayer (ASB). The top and side views of the defect-free supercell are shown in Fig. 1a, while the samples with a VT and AST of MoS_2_ layer are shown in Fig. 1b and c, respectively. The defect sites are indicated by red arrows. The samples with an ASB and a DV of MoS_2_ layer are shown in Fig. 1d and e, respectively. Planar charge density distribution around the point defects in the Au/MoS_2_ samples are shown at the bottom panel in Fig. 1.

To study the effect of defect density on the SBH, we varied the lateral size of the computational cell: the supercells of 3 × 3, 4 × 4, 5 × 5 and 6 × 6 lattice unit cells of MoS_2_ monolayer accommodated on Au (111) substrate were constructed. Since defect-free MoS_2_ monolayer (PF) and defective MoS_2_ monolayers with five different types of defects (VT, VB, DV, AST and ASB) were considered for each supercell, we generated 24 atomic supercells in total. For the defect-free MoS_2_ monolayer, we used the 3 × 3, 4 × 4, 5 × 5 and 6 × 6 supercells to verify that the SBH is not affected by sample size. The defect density (and defect concentration) per unit area in the constructed samples is given in Table 2. For 3 × 3 MoS_2_ supercell, we built Au (111) substrates containing 4, 5 and 6 Au layers. We found that the difference in the obtained SBH values was rather minor, thus for the remaining samples, we only constructed the Au (111) substrate with 4 layers. All the atom positions were relaxed except for the atoms in the two bottom layers of the Au (111) substrate which kept fixed to mimic an infinite substrate.

**Table 2:** Defect density per unit area and defect concentration for the constructed supercells.

Au/MoS_2_ sample size	Defect density per unit area (1/Å^2^)	Defect concentration (%)
6 × 6 × 4	0.003	1
5 × 5 × 4	0.005	2
4 × 4 × 4	0.008	3
3 × 3 × 4	0.014	6

Periodic boundary conditions were applied along all the directions, while a vacuum layer with the thickness of ~ 20 Å was added as a padding along the Z-direction (normal to the Au(111) surface, see Fig. 1) to avoid spurious interactions due to periodic boundary conditions. Considering that there is a lattice mismatch (~ 6%) along the lateral (X, Y) directions between the lattice constants of primitive unit cells of MoS2 monolayer and that of Au(111) surface, the metallic substrate was elongated along the lateral directions to eliminate the mismatch. This is a common practice^43,53^, which allows to apply periodic boundary conditions in DFT calculations. The physical basis for this treatment is that small deformation of the metallic substrate leads to a minor change in its electronic band structure and work function. For example, we found that the work function of the Au(111) substrate was reduced only by ~ 2% by the tensile strain. The geometry of constructed samples was optimized by DFT method using conjugate-gradient optimization.

Two DFT-based methods, that is, the method based on the projected electronic band structure and the method based on the SM rule, have been commonly used to calculate the SBH. We used both methods to calculate the SBH and to compare their reliability and accuracy. Below, we briefly discuss these methods.

### The method based on the projected electronic band structure

**Figure 2:**
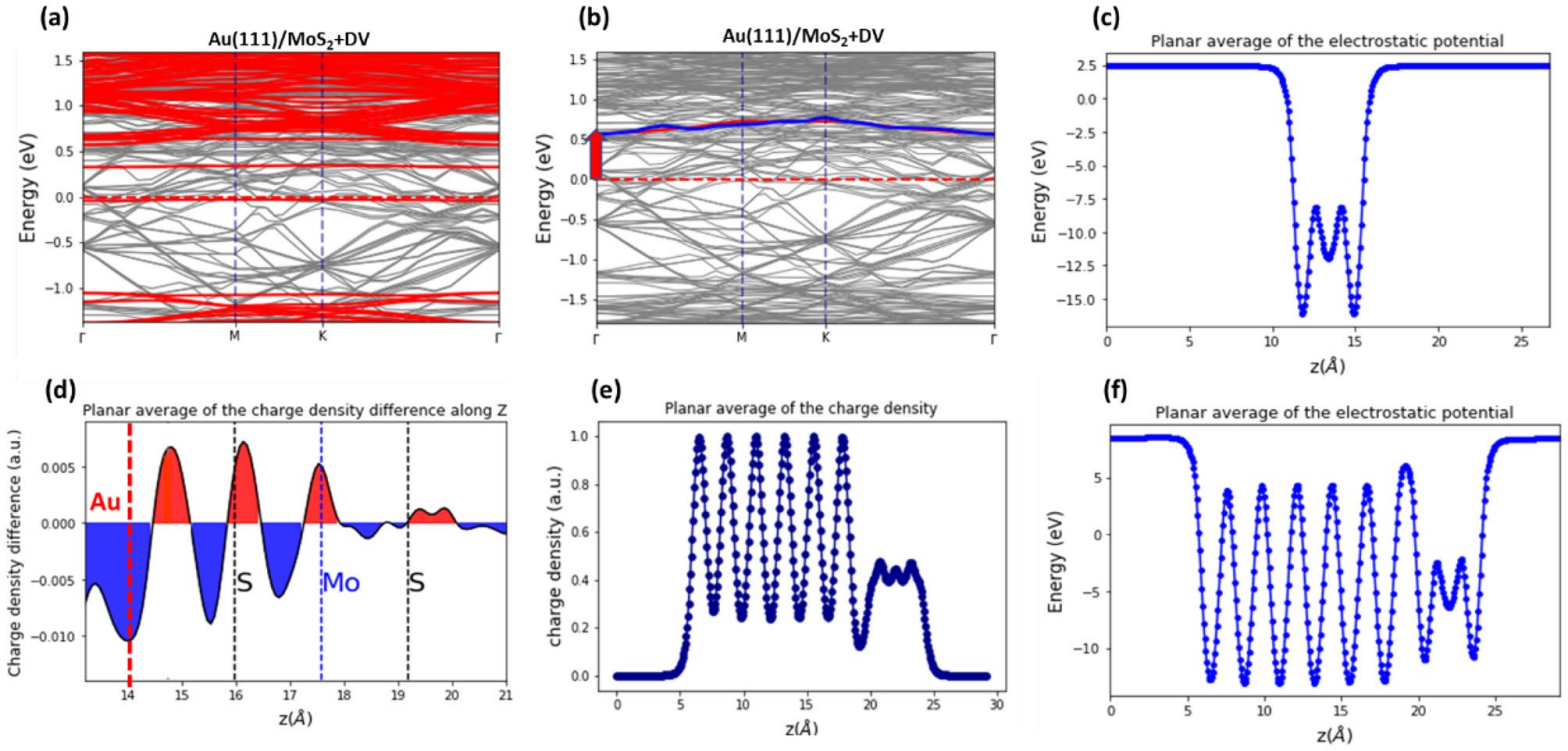
(**a**) The electronic band structure of the MoS_2_ monolayer with a double sulfur vacancy (red bands) superimposed over the band structure of Au/MoS_2_ heterojunction (grey bands). (**b**) The superimposed CBM band of MoS_2_ monolayer containing a double sulfur vacancy (red band) is matched in a high accuracy with one of the Au/MoS_2_ junction bands (blue band). The distance from the minimum of the matched band to the Fermi level indicates the SBH value (shown by arrow at Γ-point). (**c**) The planar average of charge density of the MoS_2_ monolayer with a double sulfur vacancy. (**d**) The planar average of charge density difference for Au/MoS_2_ junction with a double sulfur vacancy. Red color indicates the charge accumulation regions and blue the charge depletion regions. The dashed lines indicate the average Z-position of Au-atoms at the top surface layer, and S-atoms in the top and bottom MoS_2_ sublayers, as well as Mo-atoms in the middle sublayer. (**e**) The planar average of charge density of the Au/MoS_2_ heterojunction. (**f**) The planar average of Hartree (electrostatic) potential of the Au/MoS_2_ junction a double sulfur vacancy in the MoS_2_ monolayer. The Z-axis is normal to the Au(111)/MoS_2_ contact plane and the plane average is calculated over [XY] planes along the sample. The plots are for the Au(111)/MoS_2_ 3 × 3×6 sample.

In this method, the SBH is obtained by identifying the position of conduction band minimum (CBM) of the contact MoS_2_ layer amongst the bands of the Au(111)/MoS_2_ heterojunction. The SBH is the distance from the Fermi level to the identified CBM^18,37^. Hence, to calculate the SBH, one needs to obtain the electronic band structure of the Au(111)/MoS_2_ heterojunction, and that of the contact MoS_2_ monolayer taken from the Au(111)/MoS_2_ heterojunction. In the case when a free-standing MoS_2_ layer is accommodated on a substrate, its geometry, and therefore its electronic band structure is altered. Therefore, to calculate its electronic band structure in the Au(111)/MoS_2_ heterojunction, we have to take the MoS_2_ layer from the heterojunction (while keeping all the atomic positions fixed). By using this MoS_2_ monolayer with the fixed contact geometry, one can obtain its CBM accurately (see the red-colored band structure in Fig. 2a).

Next, the electronic band structure of contact MoS_2_ layer is projected onto the electronic band structure of Au(111)/MoS_2_ heterojunction (see Fig. 2a). When the superimposed electronic bands align, one can identify an electronic band of the Au(111)/MoS_2_ heterojunction overlapping with the CMB band of the contact MoS_2_ monolayer. The two overlapping bands have almost identical shapes. In Fig. 2b, the red-colored bottom conduction band (CBM) of MoS_2_ contact monolayer matches one of the electronic bands of the Au(111)/MoS_2_ heterojunction (see the blue line in Fig. 2b). The distance from the Fermi level to the minimum of the identified band (which is indicated by the red arrow in Fig. 2b) is equal to the SBH of the of Au(111)/MoS_2_ heterojunction^11,18,40^.

The MoS_2_ monolayer-substrate interaction places a particular limitation on application of this approach: the interaction must be comparatively weak so that the band structure of MoS_2_ is perturbed only to a small extent. This method is applicable to the Au/MoS_2_ heterojunction, since the interfacial bonding is attributed to relatively weak van der Waals interaction^18,40^. Therefore, we applied this method for both defect-free and defective MoS_2_ monolayer. So far as known, point defects introduce new defect states in the band gap of MoS_2_ monolayer: occupied defect states below the Fermi level and unoccupied ones above it. Since the vacancy produces localized states^54^, we used the CBM position (as defined for defect-free monolayer) to obtain the SBH.

In addition to the electronic band structure, partial density of states (pDOS) is a convenient way to illustrate the effect of point defects in the MoS_2_ layer on the electronic properties of Au/MoS_2_ junction (see Figs. 4, 5 and 6). The pDOS is calculated separately for the Mo- and S-atoms of the contact layer as an average over all the atoms and their corresponding orbitals (five 4d-orbitals for Mo-atoms and three 3p-orbitals for S-atoms). The position of CBM cannot be identified from the pDOS plots with high accuracy since the band edge shape in pDOS plot is often fuzzy. The exact position was taken by using the method based on the projected electronic band structure.

### The method based on the SM rule

Another commonly used method to calculate the SBH is based on the SM rule^15^. According to this rule, the value of SBH for a metal/semiconductor junction is proportional to the difference of metal work function, ${W}_{m}$, and the semiconductor EAE, $\chi$: $\Phi ={W}_{m}-\chi .$ For a metal, which is in our case Au(111) substrate, the work function is defined as the difference between its vacuum energy level and the Fermi energy. We obtained ${W}_{Au}$ = 5.11 eV from our DFT calculations with PBE XC-functional, and ${W}_{Au}$ = 5.27 eV with PBE XC-functional and DFT-D2 van der Waals correction. It is noted that the calculated values are slightly lower than previously reported values of ${W}_{Au}$ = 5.13 eV and ${W}_{Au}$ = 5.3 eV^11,18^, since we deformed the Au(111) sample to eliminate the lattice mismatch between Au(111) and MoS_2_ monolayer to enable the application of periodical boundary conditions.

The EAE, denoted as ${\chi }_{{MoS}_{2}}$, is calculated as the difference between the vacuum energy level (obtained as an asymptotic value of planar averaged electrostatic potential, which is taken sufficiently far off the monolayer, see Fig. 2c) and the energy level of the CBM, which is identified by using the calculated electronic band structure of the MoS_2_ layer. In our case, the ${\chi }_{{MoS}_{2}}$ varies within a certain range around ${\chi }_{{MoS}_{2}}=4.21 eV$ for defective monolayer (see Fig. 7b and Tables S1–S4 in Supplementary Materials).

To account for the interaction between the MoS_2_ monolayer and the underlying metallic substrate, and for the corresponding change in the work function of the substrate in the presence of MoS_2_ monolayer, the original SM rule^15^ must be modified. When the MoS_2_ monolayer and Au substrate are integrated into the Au/MoS_2_ heterojunction, the equalization of the Fermi levels results in the charge transfer from the gold substrate to the MoS_2_ monolayer (see Fig. 2d, where the charge accumulation and depletion zones at the Au(111)/MoS_2_ heterojunction are exemplified), which alters the SBH. The charge transfer and its redistribution at the Au/MoS_2_ heterojunction results in the potential step, $\Delta V$, given by $\Delta V=\frac{{e}^{2}}{A}\iiint z\Delta n(x,y,z)dxdydz$, where $A$ is the contact area (measured within the [X,Y] plane), and $\Delta n(x,y,z)={n}_{Au/{MoS}_{2}}(x,y,z)-{n}_{Au}(x,y,z)-{n}_{{MoS}_{2}}(x,y,z)$ is the difference between the electronic density of Au(111)/MoS_2_ interface, ${n}_{Au/{MoS}_{2}}$, (which is illustrated in Fig. 2e for the Au(111)/MoS_2_ heterojunction containing double S-vacancies) and the electronic density of Au substrate, ${n}_{Au}\left(x,y,z\right)$ and that of MoS_2_ monolayer, ${n}_{{MoS}_{2}}\left(x,y,z\right).$ According to the modified SM rule, which includes the effect of the interface potential step, the SBH value is given by: ${\Phi }_{Au/{MoS}_{2}}={W}_{m}$-${\chi }_{{MoS}_{2}}$−$\Delta V$^18,40^. The interface potential step is attributed to the reduction in the metal work function due to its contact with the MoS_2_ monolayer. The change in the work function is a combined effect of the rehybridization of d-orbitals of Au-atoms^13^, polarization of the metal electrons induced by the MoS_2_ monolayer^55^, the “pushback” effect (the displacement of surface electron density around the metallic substrate into the metal by the MoS_2_ monolayer^56^), the Pauli repulsion at the interface (which is the main contribution to the interface potential step in the weakly interacting regime^40,57,58^), the presence of interface dipole moment^18^ and the surface relaxation of metallic substrate^37,40^.

The potential step at the interface can be calculated either by using the planar average electronic charge density along the z-direction, $n$(z), or by using the plane-averaged electrostatic (Hartree) potential defined as $V(z)=\frac{{e}^{2}}{A}\iint z\Delta n\left(x,y,z\right)dxdy.$ According to Farmanbar et al.^40^, the potential step can be obtained by inspecting the asymptotic values of $V\left(z\right)$ for the Au/MoS_2_ junction in the vacuum, which are typically attained within a few Å from the metallic surface at the bottom and within a few Å from the MoS_2_ layer at the top (see Fig. 2f, where the plane-averaged electrostatic potential is shown for the Au(111)/MoS_2_ junction with double S-vacancies). Thus, one can calculate the value of $\Delta V$ as the difference of $V(z)$ taken between two points located at sufficiently large distance deep in the vacuum (at the points where electrostatic potential $V\left(z\right)$ converges to constant values). Since the periodic boundary conditions are applied in the DFT calculations, one needs to use dipole corrections along the z-axis to obtain the well-defined potential step in $V\left(z\right).$

### Comparison of the two methods

**Figure 3:**
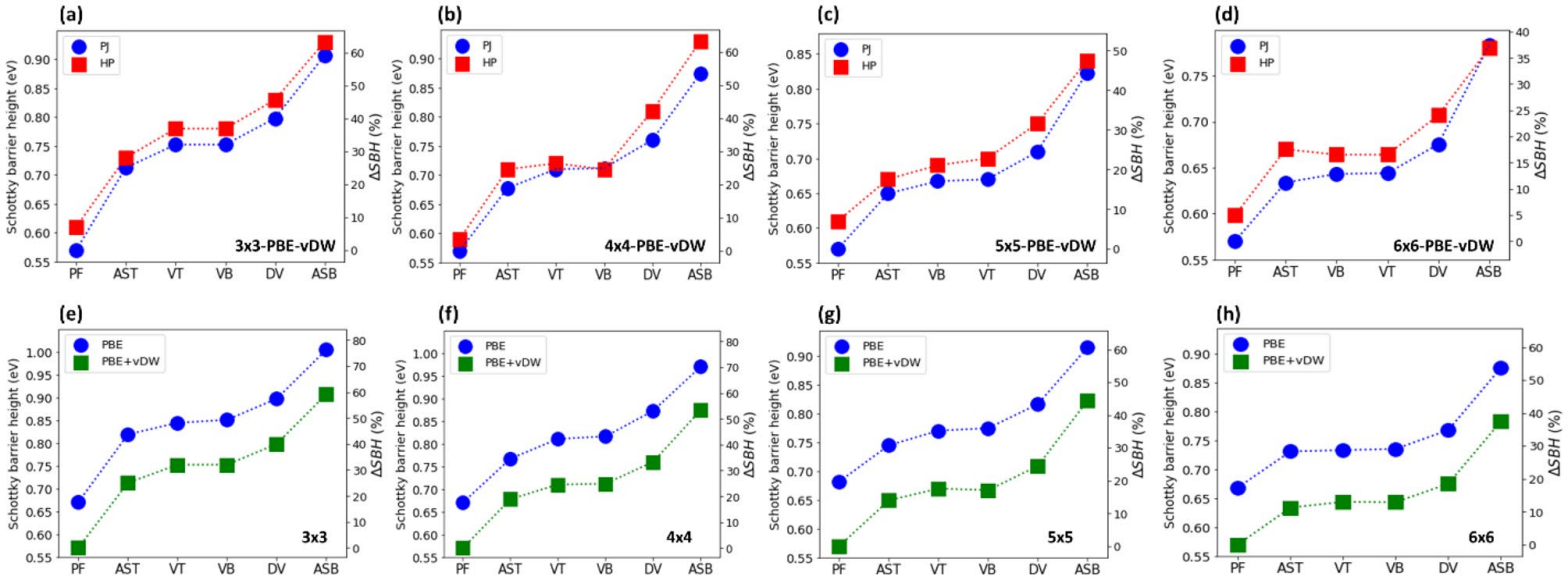
(**a**) Comparison of the SBHs calculated with the method based on the projected (PJ) electronic band structure (blue circles) and the method based on the modified SM rule using Hartree electrostatic potential (HP, red squares). On the left vertical axis, the SBH for Au (111)/MoS_2_ junction with PF, VT, VB, DV, AST, and ASB. The data is for the 3 × 3 (with defect density $${n}_{d}$$ = 14 × 10^–3^ Å^-2^), 4 × 4 ($${n}_{d} $$= 8 × 10^–3^ Å^-2^), 5 × 5 ($${n}_{d}$$ = 5 × 10^–3^ Å^-2^) and 6 × 6 ($${n}_{d}$$ = 3 × 10^–3^ Å^-2^) Au(111)/MoS_2_ junctions. On the right axis, the relative increase of the SBH with respect to the defect free sample $$\Delta SBH\left(\%\right)=100 \times \left(\frac{SBH-{SBH}_{0}}{{SBH}_{0}}\right)$$. (**b**, **c**) Comparison of the SBHs calculated with the PBE exchange–correlation (XC) potential (blue circles) and PBE-XC and van der Waals DFT-D2 corrections (green squares) for Au(111)/MoS_2_ junctions. The defect density is the same as in (**a**).

To compare these two methods, we plot the calculated SBH values obtained by using the band structure projection (see blue circles in Fig. 3a–d) and by using the SM rule (see red squares in Fig. 3a–d). The results show a remarkably similar trend between the obtained SBH, and the defect type as shown in Fig. 3a–d (see also Tables S1–S4 and Fig. S5 in Supplementary materials). On average, the difference in the SBH values obtained by these methods is $\sim$ 3%, while the maximal difference is $\sim$ 7%. We note that the difference in the SBH values obtained by the two methods in this study are smaller than previously reported^40^.

### Details of DFT calculations

All our calculations were carried out by using density functional theory (DFT) with the generalized Perdew-Burke-Ernzerhof^59^ and the projector-augmented wave (PAW) pseudopotential plane-wave method^60^ for the core electrons as implemented in the Vienna ab initio simulation package (VASP) code^61^. For the PAW pseudopotentials, we included 5d^10^6s^1^, 4d^5^5s^1^, and 3s^2^3p^4^ as valence electrons for Au, Mo, and S, respectively. For DFT calculations, we used 6 × 6 × 1 Monkhorst − Pack^62^ k-point grid for the geometry optimizations, and a plane-wave basis set with an energy cut-off of 520 eV was adopted. Good convergence was obtained with these parameters, and the total energy was converged to 10^−7^ eV per atom. The atomic samples were fully relaxed with a residual force of less than 0.02 eV/Å. Spin polarization was considered in this study. The energy minimization was performed using a conjugate-gradient algorithm to relax the ions into their instantaneous ground state. The DFT calculations were done with van der Waals corrections using Grimme’s DFT-D2 approach as realized in the VASP^61^. Dipole corrections to the total energy were used along the direction normal to Au(111)/MoS_2_ interface for all the calculations^63^.

We note that the application of van der Waals corrections not only leads to more accurate results, but it is crucial for Au substrate. In contrast to other more reactive metallic surface like Mo and Ti, where bonds are formed^13^, the van der Waals nature of the Au − MoS_2_ interaction is prevalent. Covalent bonds between Au and S atoms cannot be formed since the Au atom with one s-electron has fully occupied d-orbitals, and hence only weakly interacts with MoS_2_^40^. In Fig. 3e–h, we compare the SBH calculated with (green squares) and without (blue circles) van der Waals corrections. It is evident that application of van der Waals corrections systematically lowers the SBH values by ~ 15% (~ 0.1 eV).

### Beyond PBE functional: hybrid HSE XC-potential

There is an uncertainty in the calculated SBHs coming from using PBE functional. In our DFT calculations, we used the PBE functional, but it is well-known that it underestimates the band gap of MoS_2_ since it does not take into account the many body effect among electrons, only partially accounts for electronic correlation, and neglects long-range exchange and subtle screening effects^24,64^. We obtained a direct band gap of ${E}_{g}$ = 1.7 eV for MoS_2_ monolayer using PBE with DFT-D2 van der Waals corrections, which is in a good agreement with previous GGA calculations^11,20^, while calculations based on the GW-quasiparticle approximation give ${E}_{g}$ = 2.8 eV^65,66^ and application of hybrid HSE XC-potential results in ${E}_{g}$ = 2.2 eV^26^. Even though the electronic band gap for free-standing MoS_2_ monolayer is not well-known, the results obtained with HSE and GW-quasiparticle approximations are in excellent agreement with the experimentally measured optical band gap is ${E}_{g}$ = 2.9 eV^21,67^. It must be admitted that the electronic band gap is fundamentally different from the optical gap, which is generally measured by photoluminescence experiments^68^. The optical band gap corresponds to the energy required to create an exciton, while the electronic band gap also requires the breaking of the exciton, and is thus higher due to the exciton binding energy. Exciton binding energies between 0.01 and 0.5 eV have been reported^40^. A direct comparison of the PBE vs. GW-quasiparticle approximation is not truly fair, as the observed difference includes the exciton binding energy, obtained by using the GW-quasiparticle approximation.

We estimated the required corrections when the hybrid density XC-functional potential is applied. Hybrid functionals mix a fraction of the short-range part of the Hartree–Fock (HF) exchange interaction with the local functional. There is a range of hybrid functionals, among them, we selected the Heyd, Scuseria, and Ernzerhof (HSE) hybrid functional^69^. This hybrid density functional is based on a screened Coulomb potential for the exchange interaction which circumvents the bottleneck of calculating the exact (Hartree–Fock) exchange, especially for systems with metallic characteristics. The main reason for the selection is due to its high accuracy combined with its computational advantages for periodic systems^69^. Moreover, the conduction band in MoS_2_ consists of d-orbitals and PBE functional has significant limitations in proper description of localized d-electron states. Therefore, we complement our DFT calculations with hybrid functional calculations for the band structures, Hartree potential and defect states.

### The effect of spin–orbit coupling

It is well-known that spin–orbit coupling (SOC) is large in the valence band of MoS_2_ monolayer^70,71^, thus it may affect the SBH. To verify the effect of SOP on the SBH, we carried out DFT calculation with SOC for the defect-free and defective 4 × 4 × 4 and 5 × 5 × 4 samples in which S monovacancy, VB, and Mo_S_ antisite defect, ASB, located at the bottom sublayer of MoS_2_ monolayer were considered. It was found that SOP does not affect the SBH in all the considered cases, which agrees with the results obtained by Szczȩśniak et al.^72^ and Chen et al.^73^ for defect-free metal/TMD heterojunctions.

## Results and discussions

### Defect-free sample

**Figure 4:**
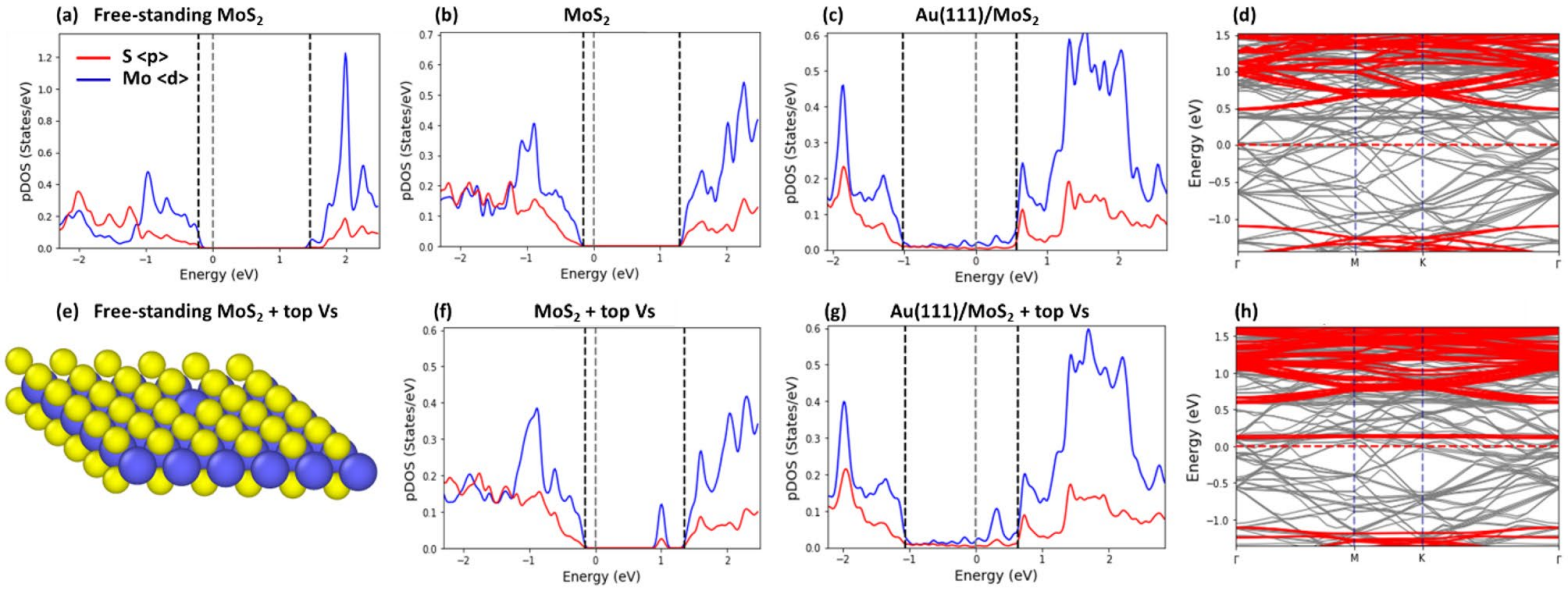
The partial density of states (pDOS) of a free-standing defect-free MoS_2_ layer (**a**) and the contact MoS_2_ layer (**b**) taken from the Au/MoS_2_ heterojunction. The pDOS of the Au/MoS_2_ sample calculated as an average over d-orbitals of Mo-atoms indicated by blue, and over p-orbitals of S-atoms indicated by red is shown in (**c**). The valence band maximum (VBM), Fermi level and conduction band minimum (CBM), obtained with the projection method are shown by dashed lines. (**d**) The electronic band structure of the contact layer (red bands) superimposed over the band structure of Au/MoS2 heterojunction (grey bands). (**e**) The contact MoS_2_ layer containing a top sulfur monovacancy taken from the respective Au/MoS_2_ junction. (**f**, **g**) The pDOS of the contact layer (**f**) and Au/MoS_2_ heterojunction (**g**). (**h**) The electronic band structure of the contact layer with a VT defect (red bands) superimposed over the band structure of the Au/MoS_2_ junction (grey bands). The sample size is 6 × 6 × 4 with PBE XC + van der Waals DFT-D2 corrections.

First, we examined the effect of different types of point defects on the SBH. To investigate this effect, we used the Au(111)/MoS_2_ junction with a defect-free MoS_2_ monolayer as a reference, which was compared with the samples containing defects. In Fig. 4a, we plot the pDOS of a defect-free free-standing MoS_2_ monolayer, which was calculated as an average over 4d-orbitals of Mo-atoms and 3p orbitals of S-atoms. For comparison, the pDOS of the MoS_2_ contact layer taken from the Au(111)/MoS_2_ heterojunction is shown in Fig. 4b. It should be readily seen that the rearrangement of atomic position in the contact layer due to its interaction with the underlying substrate changes the overall shape of pDOS, but the band gap and the location of the CBM are nearly the same.

The pDOS of the Au(111)/MoS_2_ heterojunction and the corresponding electronic band structure are shown in Fig. 4c and d, respectively. In addition, we project the electronic band structure of the contact MoS_2_ layer onto the Au (111)/MoS_2_ band structure (see the red-colored electronic bands in Fig. 4d). The electronic band structure and pDOS are apparently changed due to the interaction of the MoS_2_ layer with the underlying Au(111) substrate.

The mid-gap states appear in the band gap of MoS_2_ monolayer as shown in Fig. 4c. Direct orbital hybridization occurs between Au- and S-atoms at the Au/MoS_2_ interface due to the overlap of their wave functions, while S-atoms mediate indirect orbital hybridization between Au- and Mo-atoms, resulting in formation of mid-gap states^11^. The Fermi level at the interface, which determines the SBH, is now governed by the charge transfer and filling of the mid-gap states. Although the density of mid-gap states is somewhat low, it is sufficient to pin the Fermi level above the middle of MoS_2_ band gap, as in an n-type contact^17,22^. Fermi pinning sets the Fermi level close to the MoS_2_ CBM, preventing from reaching it.

The position of MoS_2_ CBM in the pDOS of the Au(111)/MoS_2_ contact is indicated in Fig. 4c. If one compares the pDOS of the free-standing MoS_2_ monolayer in Fig. 4a, b with the pDOS of the Au(111)/MoS_2_ sample, it is apparent that the position of the CBM edge, which determines the SBH value, is shifted closer to the Fermi level. To accurately pinpoint the CBM edge location, we use the projection method as shown in Fig. 4d, which is consistent with the pDOS estimation. We note that the CBM is located at the Γ point in accordance with previous reports^8,18,40^. The SBH value for the Au(111)/MoS_2_ contact sample with defect-free monolayer obtained with the SM rule based method is reported in Supplementary materials.

### S monovacancies

Next, VT and VB defects are introduced in the MoS_2_ monolayer (see Fig. 4e). An introduction of S monovacancy creates dangling bonds in the neighboring Mo-atoms, which lead to a defect state in the band gap positioned close to the bottom of conduction band (see also the distinct peak in the pDOS of a free-standing MoS_2_ monolayer in Fig. 4f). The new defective state is mainly due to the dominant 4d-states of Mo-atoms with only a small mixture of 3p states of S-atoms.

**Figure 5:**
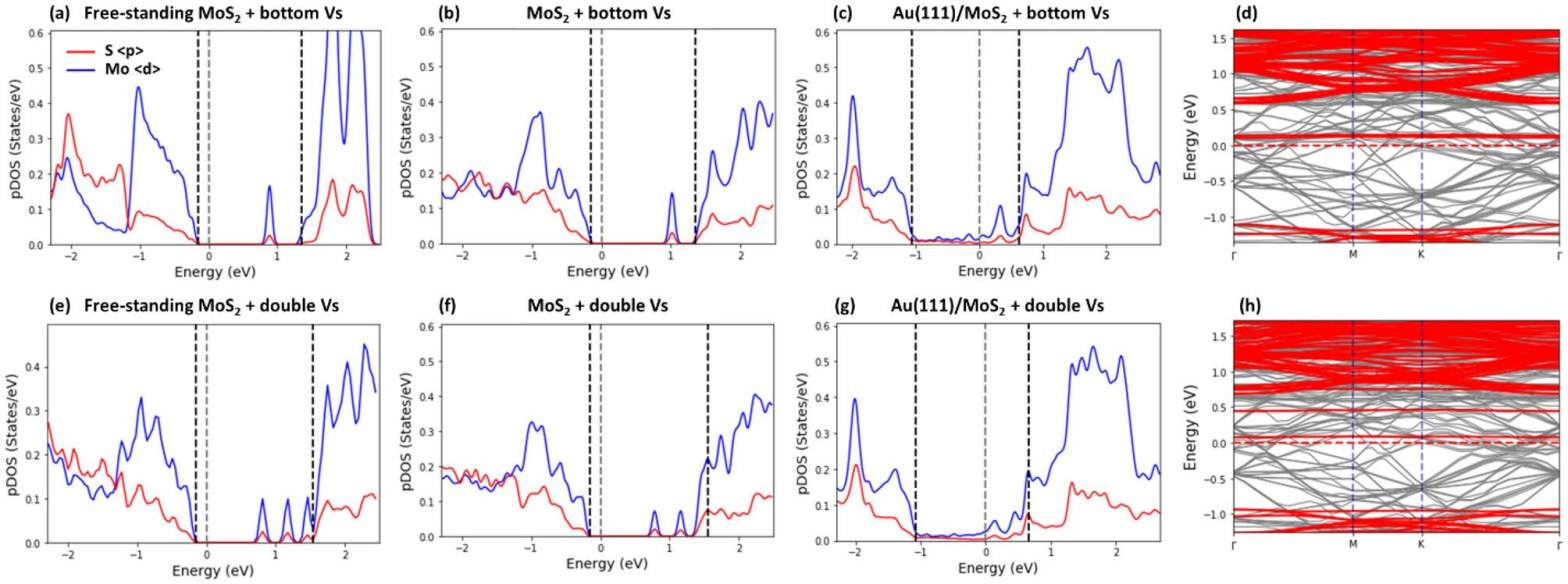
(**a**–**c**) The pDOS of a free-standing MoS_2_ layer with a single sulfur vacancy (**a**) and the contact MoS_2_ layer with a VB defect (**b**) taken from the respective Au/MoS_2_ contact. The pDOS of the Au/MoS_2_ heterojunction is shown in (**c**). (**d**) The electronic band structure of the contact layer (red bands) superimposed over the band structure of Au/MoS2 sample (grey bands). (e–g) The pDOS of a free-standing MoS_2_ layer with a DV defect (**e**) and the contact MoS_2_ layer with a DV defect (**f**) taken from the corresponding Au/MoS_2_ heterojunction. The pDOS of the Au/MoS_2_ sample is shown in (**g**). (**h**) the electronic band structure of the contact layer (red bands) superimposed over the band structure of Au/MoS_2_ sample (grey bands). The sample size is 6 × 6 × 4, for PBE XC + van der Waals DFT-D2 corrections.

The pDOS for atoms in the MoS_2_ layer taken from the Au(111)/MoS_2_ sample is fairly similar to that of the free-standing MoS_2_ layer (see Fig. 4f for VT defect and Fig. 5b for the VB defect). However, due to the interaction of MoS_2_ layer with its underlying substrate, the position of the peak in the band gap corresponding to the defect state shifts closer to the bottom of conduction band.

In the pDOS for Mo- and S-atoms of the Au(111)/MoS_2_ sample with the MoS_2_ layer containing S monovacancies (see Fig. 4g for the VT defect and Fig. 5c for the VB defect), new states (with small density amplitude) appear in the band gap due to the mixing and hybridization of S-atom orbitals, and to some extent Mo-atom orbitals with the orbitals of surface Au-atoms. The height of the vacancy-related peak somewhat diminishes, while its width broadens. We used the projection method based on the electronic band structure of the Au(111)/MoS_2_ samples to identify the CBM positions (see Fig. 4h for the VT defect and Fig. 5d for the VB defect, respectively), and found that the SBH increases in the range from $\sim$ 10 to $\sim$ 30% due to the S monovacancies. We found that the SBH also depends on the number of S monovacancies per unit area, which will be discussed later.

### S divacancies

Next, we calculated the pDOS for S- and Mo-atoms of a free-standing MoS_2_ monolayer with S divacancies, which were created by removing S-atoms from both the top and bottom sublayers of MoS_2_ layer as shown in Fig. 1e. The pDOS results are shown in Fig. 5e. It is seen that S divacancies result in the three distinct peaks in the band gap located above the Fermi level, with one of them being near the bottom of the conduction band.

The pDOS for S- and Mo-atoms of the MoS_2_ monolayer with S divacancies taken from the Au(111)/MoS_2_ sample is shown on Fig. 5f.The rearrangement in the atomic positions of the defective MoS_2_ (due to its interaction with Au(111) substrate) modifies the pDOS, particularly the shape of peak in the proximity to the bottom of conduction band.

In Fig. 5g, we plot the pDOS for S- and Mo-atoms of the MoS_2_ layer with S divacancies accommodated on Au(111) surface. The interaction of the defective MoS_2_ layer with the Au substrate changes its pDOS substantially. As can be seen in Fig. 5g, new states, with a low-density amplitude, appear in the band gap around the two divacancy-related peaks. The peaks merge to some extent, forming a double hump shape, while the third peak merges with the bottom of the conduction band.

The applications of the method based on projection of the electronic band structure (see Fig. 5h) and the method based on the SM rule show that in the presence of S divacancies, the SBH increases by $\sim$ 20–40% as compared to that of the Au (111)/MoS_2_ contact sample with a defect-free monolayer. The effect of S divacancy is almost twice as larger as that of S monovacancy, and thus can be approximately considered as a linear superposition of two monovacancies.

### Antisite defects

**Figure 6:**
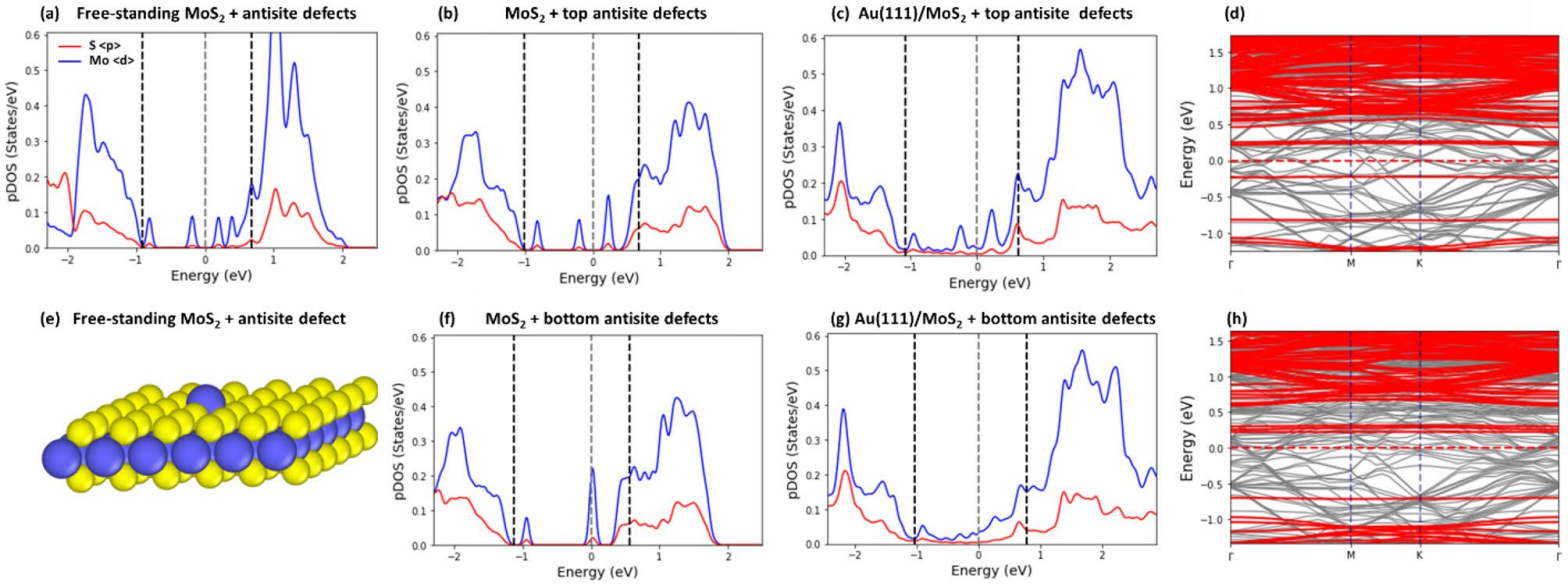
(**a**–**c**) The pDOS of a free-standing MoS_2_ layer with a single antisite defect (a) and the contact MoS_2_ layer with an AST defect (**b**) taken from the Au/MoS_2_ heterojunction. The pDOS of the Au/MoS_2_ sample is shown in (**c**). (**d**) The electronic band structure of the contact layer (red bands) superimposed over the band structure of Au/MoS2 sample (grey bands). (**e**) The geometry of the contact layer with an AST defect (**f**). The pDOS of the contact MoS_2_ layer with an ASB defect (f) taken from the corresponding Au/MoS_2_ junction. The pDOS of the Au/MoS_2_ sample is shown in (**g**). (**h**) The electronic band structure of the contact layer (red bands) superimposed over the band structure of Au/MoS_2_ heterojunction (grey bands). The sample size is 6 × 6 × 4 with PBE XC + van der Waals corrections.

The effect of antisite defects introduced in the top (AST) or bottom (ASB) sublayer of MoS_2_ layer (see Fig. 1c, d) on the pDOS, band structure and SBH of the Au(111)/MoS_2_ sample are illustrated in Fig. 6. It is seen that antisite defects markedly change the pDOS of a free-standing MoS_2_ monolayer (see Fig. 6e). Five localized defect states occur within the band gap of the free-standing MoS_2_: Two states below the Fermi level and three above it, with one being in the vicinity of the bottom of conduction band (see Fig. 6a).

When the MoS_2_ layer is placed on an Au(111) substrate, its electronic structure changes significantly as the result of its interaction with the underlying gold surface. In Fig. 6b, we plot the pDOS for atoms of MoS_2_ layer with an AST defect taken from the Au (111)/MoS_2_ heterojunction (see also Fig. 6f for MoS_2_ with an ASB defect). The geometry and the corresponding pDOS of the contact MoS_2_ layer are markedly modified by the underlying substrate: the two peaks of a free-standing layer located above the Fermi level now merge into a single peak for the MoS_2_ with an AST defect (see also the corresponding band structure in Fig. 6h). In contrast, three peaks located near the Fermi level now merge into one for the MoS_2_ with an ASB defect (see Fig. 6f and the corresponding band structure in Fig. 6h). Contrary to the monovacancy defects, the difference in the pDOS between the AST and ASB defect in the MoS_2_ monolayer is considerably larger.

Even more revealing is the change in the pDOS for S- and Mo-atoms of the MoS_2_ monolayer with the ASB defects. As can be seen in Fig. 6c, in the case of MoS_2_ layer containing AST defects, the two defect states located above the Fermi level merge into one, while many additional states appear around it within the band gap. However, the shape of pDOS resembles that of the contact monolayer (or the free-standing monolayer). In the case of MoS_2_ layer containing the ASB defects, the changes in the pDOS are substantial as compared with the contact (or free-standing) MoS_2_ layer (see Fig. 6g). The different defect-related peaks merge with the new states within the band gap and form a broad continuum. This indicates that the interaction of the MoS_2_ layer with the ASB defects is stronger than that with the AST defects. We calculated and compared the relative changes in the binding energy and the interfacial distance between the defective MoS_2_ monolayer and its defect-free counterpart and found that the effect of ASB defects is noticeably stronger than that of AST defects (see Fig. S7 in Supplementary materials).

The methods based on the projection of electronic band structure (see Fig. 6d and h) and on the SM rule were applied to calculate SBH for MoS_2_ layer with antisite defects. It was found that the presence of AST defects increases SBH in the range from $\sim$ 10 to 25% (the SBH increases in direct proportion to antisite density). However, when ASB defects are present, the effect on the SBH is much more profound: the increase in SBH is in the range of $\sim$ 40 to $\sim$ 60% according to the number of the ASB defects per unit area.

### Comparison of different point defects

**Figure 7:**
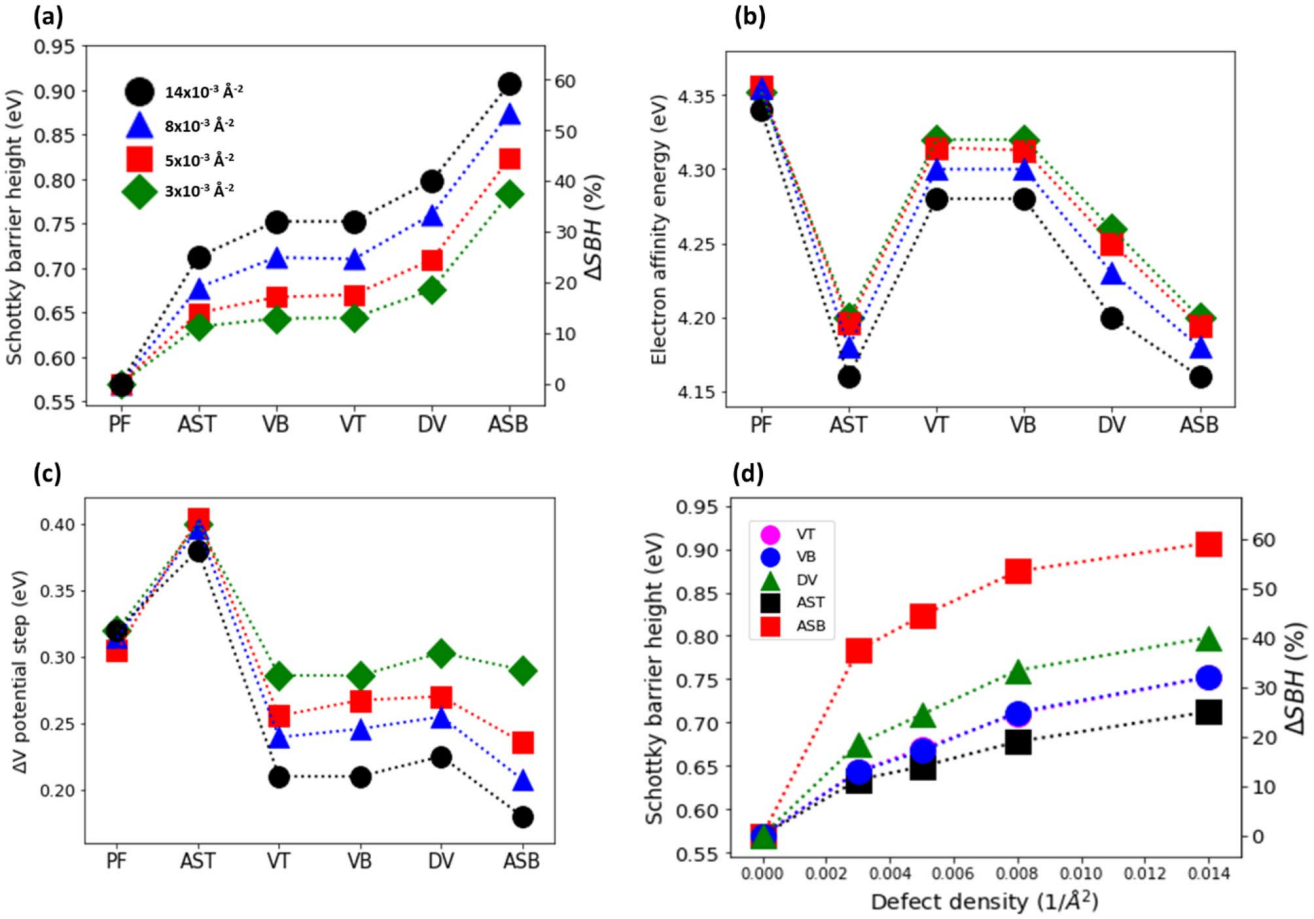
(**a**) The effect of point defects on SBH. On the left vertical axis, the SBH values for Au (111)/MoS_2_ contact with a defect-free monolayer (PF) and a monolayer containing VT, VB, DV, AST, ASB defects. The data is for 3 × 3 samples with 6 Au layers (black circles), and 5 × 5 (red squares), 4 × 4 (blue triangles), and 6 × 6 (green diamonds) Au/MoS_2_ samples with 4 Au layers. The corresponding defect densities are indicated. The right axis shows the relative increase of the SBH with respect to the defect free sample, that is, $\Delta SBH\left(\%\right)=100*\left(\frac{SBH-{SBH}_{0}}{{SBH}_{0}}\right)$. (**b**, **c**) The EAE (**b**) and potential step (**c**) of defect-free and defective MoS_2_ monolayer for Au (111)/MoS_2_ contact. (**d**) The effect of defect concentration on the SBH values for Au/MoS_2_ sample with a MoS_2_ monolayer containing VT (blue circles), VB (magenta circles), DV (green triangles), AST (black squares) and ASB (red squares) defects. The SBH value of the defect-free sample is given for comparison. Right axis shows the relative increase of the SBH with respect to the defect free sample. The DFT calculations with DT2 van der Waals (vDW) corrections are used. The SBHs values are obtained based on projection of electronic band structure.

In Fig. 7a, we summarize the obtained SBH results for Au(111)/MoS_2_ heterojunctions with a defect-free MoS_2_ monolayer, as well as MoS_2_ monolayer with VT, VB, DV, AST, and ASB defects. The impact of ASB and DV is the strongest, and that of VT and VB defects is in the middle, while that of AST defects is the weakest (see also Table S5 in Supplementary Materials).

The SBH is calculated according to SBH = ${W}_{Au}-{\chi }_{{MoS}_{2}}-\Delta V$, where ${W}_{Au}$ is the work function of Au (111), ${\chi }_{{MoS}_{2}}$ is the EAE of MoS_2_ monolayer, and $\Delta V$ is the step in the plane averaged electrostatic potential, which represents the reduction of the work function of Au substrate in contact with MoS_2_ layer. Since ${W}_{Au}$ of the substrate is fixed, the SBH increases when the value of ${\chi }_{{MoS}_{2}}$ decreases. As can be seen in Fig. 7b, the introduced point defects (especially the antisite defects and double vacancies) reduce noticeably the ${\chi }_{{MoS}_{2}}$. Besides that, the SBH increases when the $\Delta V$ decreases. As can be seen in Fig. 7c, the hosted point defects (except for the AST defects) reduce the ∆V. However, even for the AST defects, the effect of the reduction in the value of ${\chi }_{{MoS}_{2}}$ is stronger than that of an increase in the value of $\Delta V,$ thus the overall result is a minor increase in the SBH for these defects.

An interesting question is why the defects reduce ${\chi }_{{MoS}_{2}}$? It is known that the EAE is the energy required to transfer an electron from the bottom of the conduction band to the vacuum level. The ${\chi }_{{MoS}_{2}}$ is measured as the energy difference between the CBM and vacuum level, and since the introduced point defects move the CBM position further away from Fermi level, the energy difference (and the corresponding ${\chi }_{{MoS}_{2}}$) decreases. As can be seen in Fig. S6c, d (see Supplementary Materials), the introduction of point defects changes the electrostatic Hartree potential profile (especially in their vicinity). Since the minimum value of the Hartree potential rises, the corresponding ${\chi }_{{MoS}_{2}}$, which is required to transfer an electron from the bottom of the conduction band to the vacuum level, is reduced. Thus, in the presence of point defects, the ${\chi }_{{MoS}_{2}}$, which is considered as an average over all possible sites of MoS_2_ layer, including the defect sites, decreases. The magnitude of the effect depends on both the type of point defects and their density per unit area.

As can be seen from Fig. 7c, all the considered point defects, except for the AST defects, reduce the value of $\Delta V$ in the Au (111)/MoS_2_ heterojunctions. The degree of reduction in $\Delta V$ depends on the type of point defects, which affects the interfacial dipole moment (see Fig. 2d). The smaller is the interfacial dipole, the smaller is the $\Delta V$. We found that both the amount of charge transferred from the Au(111) substrate to the defect-free MoS_2_ and the resulting interface dipole moment are rather small, in agreement with previous studies^17,22^. The introduction of point defects further reduces the magnitude of interfacial dipole (see Fig. S6b in Supplementary Materials), and hence that of ∆V, ultimately leading to the larger SBH.

### The effect of defect density on SBH

**Figure 8:**
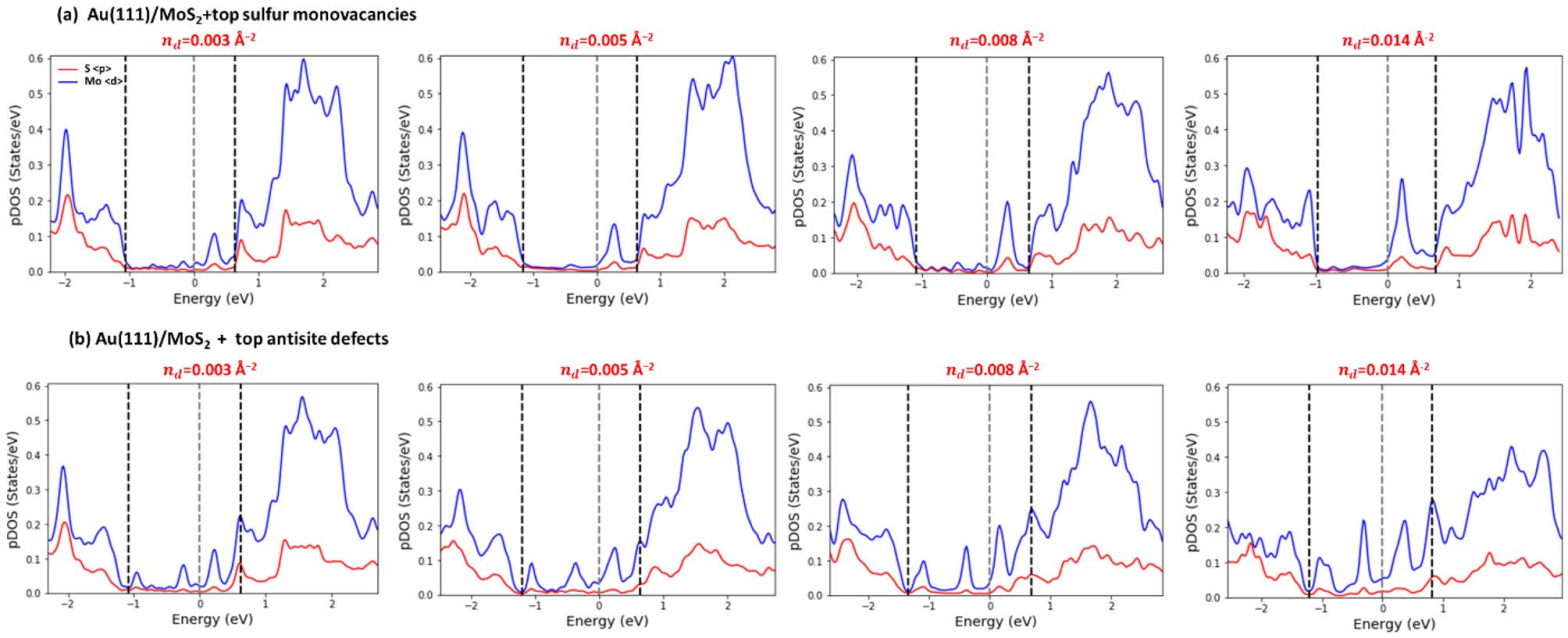
The pDOS of Au/MoS_2_ sample with the MoS_2_ monolayer containing VT vacancies (**a**) and AST defects (**b**) at the different defect densities. The defect density per unit area (${n}_{d}$) is indicated at the top of each subplot. The pDOS calculated as an average over five d-orbitals of Mo-atoms indicated by blue, and over three p-orbitals of S-atoms indicated by red. The VBM, Fermi level and CBM, which were obtained with the projection method, are shown by the dashed lines.

We next investigated how the SBH depends on the defect density. To illustrate the effect of defect density on the electronic structure of MoS_2_ monolayer at Au(111)/MoS_2_ heterojunction, we plot the pDOS for Mo- and S-atoms of a MoS_2_ monolayer with VT defects (see Fig. 8a) and AST defects (see Fig. 8b) at the different defect densities per unit area. As can be seen in Fig. 8a, the overall shape of pDOS for MoS_2_ monolayer with VT defects varies insignificantly with the defect density. The height of the peak within the band gap (and to some extent its width) enhances with an increase in the vacancy density. The distance between the Fermi level and CBM, which is the measure of SBH, increases proportionally with the defect density.

The pDOS for Au(111) /MoS_2_ sample with the MoS_2_ monolayer containing AST defects is shown in Fig. 8b at the different defect densities. Once more, one can see that at the different defect densities, the pDOS shape is similar: there are well-defined peaks located above and below the Fermi level in the band gap. It is evident that the height of the peaks grows, and their width broadens with the increase in defect density. The higher is the defect density, the more distorted is the pDOS. The similar changes in the pDOS are found for the DV, VB, and AST defects (see Figs. S1–S3 in Supplementary Materials).

The main effect of defect density on the SBH for these different point defects is summarized in Fig. 7d: the SBH monotonically increases with the defect density: The increase in the SBH is nearly linear for VT and AST defects, while it is strongly non-linear for ASB and DV defects, especially at high defect densities. In general, the higher the defect density, the stronger its impact on the electronic structure of the Au(111)/MoS_2_ heterojunction.

The defect densities per unit area for the 3 × 3, 4 × 4, 5 × 5, and 6 × 6 supercells are 0.014, 0.008, 0.005, and 0.003 (1/Å^2^). To further investigate the effect of defect density, DFT calculations on the SBH for a considerably larger 12 × 12 supercell with the defect density 0.001 (1/Å^2^) have also been carried out. As expected, the SBH for the VB, DV and ASB has been reduced to 0.58 eV, 0.59 eV, 0.61 eV, respectively. Hence, the calculated SBH values agree with the trends derived from the results using smaller supercells. These results also suggest that the higher the defect density, the higher the SBH.

## Discussions

Our study shows that the results obtained by the two methods predict remarkably similar trends between the SBH and defect type, and between the SBH and defect density. On average, the difference in the SBH values is $\sim$ 3%, while the maximal difference is only $\sim$ 7%. Interestingly, the difference in the SBH values obtained by these two methods in this study is smaller than previously reported^40^. This may be ascribed to two factors: first, we calculated the metal work function for the deformed Au(111) sample, and second, we used the contact MoS_2_ monolayer instead of the free-standing one to calculate the corresponding EAE. Clearly, these two improvements make the method based on the SM rule more accurate.

We note that the experimentally measured values of SBH for Au/MoS_2_ heterojunction fall in a broad range between 0.06 and 0.92 eV^1,8,38,39^. Our present study shows that the values of SBH can vary from 0.57 to 0.92 eV, depending on the type, density and location of point defects studied here. Hence, the present study can partially explain the large dispersion observed in experiments. In particular, the defect type and defect density play an important role. For example, the defect-free Au/MoS_2_ heterojunction has the SBH of 0.57 eV and while that with ASB defects at a high density can lead to the SBH of 0.92 eV.

It is understood that the MoS_2_ samples used in experiments can be quite inhomogeneous, and the type, density, and location of defects in the samples can vary to a great extent, which can result in the large scattering of the SBH values. The previous study^1^ has shown that the method used to fabricate the electrode to create the metal/MoS_2_ heterojunction can have a profound effect on the SBH. When a deposition method is used, the deposited ‘‘high energy’’ metal atoms can damage the lattice structure of MoS_2_, which can lead to the substantial chemical disorders, namely formation of numerous S and Mo vacancies, and even metallic-like defects (metallic impurities) at the interface^3,15,42^. These chemical disorders can have a profound effect on the SBH. In particular, these metallic-like defects can lead to local Ohmic contacts, and thus can significantly reduce the overall SBH at the metal/MoS_2_ junction, which might explain the very low values of SBH observed in some of the experiments. In contrast, when atomically flat metal thin films are transferred onto MoS_2_ monolayer (without direct chemical bonding) by using the damage-free electrode transfer technique^1^, the observed interface is effectively free from chemical disorder, and this leads to much higher values of the measured SBHs. Hence, defect engineering, for example, by controlling the type, location, and defect density per unit area can play an effective role in modulating the SBH.

## Conclusions

We performed first-principles calculations to investigate the effects of the type, location, and density of point defects in MoS_2_ layer on the SBH of the Au (111)/MoS_2_ junction. The values of SBH were calculated by applying two different methods: the method based on the projection of the electronic band structure and the method based on the SM rule. We found that these methods predict the same trend. With a couple of corrections, the two methods can lead to comparable values of SBH. Three types of point defects were studied: S monovacancy, S divacancy, and Mo_S_ antisite defects. For S monovacancy and antisite defects, their presence in the top sublayer and bottom sublayer is differentiated. Overall, the SBH is sensitive to the type, density, and location of point defects in the MoS_2_ layer. In general, the SBH for the defective MoS_2_ layer is universally higher than its defect-free counterpart, which will lead to a higher contact resistance and a lower electron injection efficiency. Among these defects, we found that the ASB and DV defects significantly increase the SBH, while the effect of VT, VB and AST defects is relatively weaker. Furthermore, the SBH monotonically increases with the defect density initially but gradually slows down. The effect of defect density for VT, VB and AST defects is smaller than that for ASB and DV defects.

The present work suggests that the reported dispersion of the experimentally measured SBH for Au/MoS_2_ junction can be at least partially accounted by the existence of point defects in MoS_2_ monolayer. The present study also suggests that the SBH can be modulated via defect engineering of MoS_2_ layer, for example, by controlling the type, location, or density of defects. Hence, our findings can serve as a guide for the control and optimization of the SBH in Au/MoS_2_ heterojunctions.

## Supplementary Information

Supplementary Information.

## Data Availability

Most of the data generated or analyzed during this study are included in this published article and its Supplementary Materials file. The remaining data used and analyzed during the current study is readily available from the corresponding author on reasonable request.
