# Author Correction: The effects of point defect type, location, and density on the Schottky barrier height of Au/MoS_2_ heterojunction: a first-principles study

**DOI:** 10.1038/s41598-022-25621-4

**Published:** 2022-12-06

**Authors:** Viacheslav Sorkin, Hangbo Zhou, Zhi Gen Yu, Kah-Wee Ang, Yong-Wei Zhang

**Affiliations:** 1 Institute of High-Performance Computing, A*STAR, 1 Fusionopolis Way, Singapore, 138632 Singapore; 2 Department of Electrical and Computer Engineering, National University of Singapore, 4 Engineering Drive 3, Singapore, 117583 Singapore; 3 Institute of Materials Research and Engineering, A*STAR, 2 Fusionopolis Way, Singapore, 138634 Singapore

Correction to: *Scientific Reports* 10.1038/s41598-022-22913-7, published online 26 October 2022

In the original version of this Article, Kah‑Wee Ang and Yong‑Wei Zhang were omitted as corresponding authors. Correspondence and requests for materials should also be addressed to kahwee.ang@nus.edu.sg and zhangyw@ihpc.a-star.edu.sg

The original Article has been corrected.

