# Supplementary material for: The effects of point defect type, location, and density on the Schottky barrier height of Au/MoS2 heterojunction: a first-principles study
Source: Sci Rep. 2022 Oct 26;12:18001. doi: 10.1038/s41598-022-22913-7 (PMC9606307; doi:10.1038/s41598-022-22913-7)
Supplement: Supplementary file 1 — Supplementary Information. [file 41598_2022_22913_MOESM1_ESM.docx]

# **Supplementary Materials**

# **The effects of point defect type, location, and density on the Schottky barrier height of Au/MoS2 heterojunction: A first-principles study**

Viacheslav Sorkin^1,^^[[1]](#footnote-1)^, Hangbo Zhou^1^, Zhi Gen Yu^1^, Kah-Wee Ang^2,3^,^[[2]](#footnote-2)^, Yong-Wei Zhang^1^,^[[3]](#footnote-3)^

# ^1^Institute of High-Performance Computing, A*STAR, 1 Fusionopolis Way, Singapore 138632

# ^2^Department of Electrical and Computer Engineering, National University of Singapore, 4 Engineering Drive 3, Singapore, 117583

# ^3^Institute of Materials Research and Engineering, A*STAR, 2 Fusionopolis Way, Singapore, 138634

# Data from DFT calculations

In this section, we report the data obtained from our DFT calculations with the method based on the projection of electronic band structure and the modified SM rule. The calculated values of Schottky barrier height (SBH), electron affinity energy (EAE), and potential step, ∆V, for Au(111)/MoS_2_ heterojunction with a defect-free monolayer as well as MoS_2_ layer with top and bottom single S-vacancies, double S-vacancies, top and bottom anti-site Mo_S_ defects are reported in Tables S1-S4. The data are obtained by using PBE exchange-correlation functional with and without van der Waals DFT-D2 corrections. The obtained data are presented in Table S1 for the 6x6x4 Au(111)/MoS_2_ sample, those in Table S2 for the 6x5x4 Au(111)/MoS_2_ sample, those in Table S3 for the 4x4x4 Au(111)/MoS_2_ sample, and those in Table S4 for the 3x3x6 Au(111)/MoS_2_ sample.

Table S1: The calculated values of Schottky barrier height (SBH), electron affinity energy (EAE), and potential step, ∆V, for Au(111)/MoS_2_ contact with a defect-free monolayer (PF) and one containing top (VT) and bottom (VB) single S-vacancies, double S-vacancies (DV), as well as top (ST) and bottom (SB) anti-site Mo_S_ defects are reported. The SBH are calculated by the method based on electronic band structure projection (SBH-PJ) and modified Schottky-Mott rule (SBH-HP). The data for the 6x6x4 Au(111)/MoS_2_ samples are calculated by using PBE exchange-correlation functional with and without van der Waals DFT-D2 corrections.

| **Defect type** | **PBE** | | | | | **PBE + van der Waals corrections** | | | | |
| --- | --- | --- | --- | --- | --- | --- | --- | --- | --- | --- |
|  | **SBH-PJ (eV)** | **SBH-HP (eV)** | **Difference (%)** | **EAE (eV)** | **∆V (eV)** | **SBH-PJ (eV)** | **SBH-HP (eV)** | **Difference (%)** | **EAE (eV)** | **∆V (eV)** |
| **PF** | **0.67** | **0.69** | **3** | **4.20** | **0.21** | **0.57** | **0.60** | **5** | **4.35** | **0.32** |
| **VT** | **0.73** | **0.76** | **4** | **4.16** | **0.18** | **0.64** | **0.66** | **3** | **4.32** | **0.29** |
| **VB** | **0.74** | **0.75** | **3** | **4.16** | **0.19** | **0.64** | **0.66** | **3** | **4.32** | **0.29** |
| **DV** | **0.77** | **0.78** | **2** | **4.14** | **0.18** | **0.68** | **0.71** | **5** | **4.26** | **0.30** |
| **AST** | **0.73** | **0.75** | **2** | **4.06** | **0.30** | **0.63** | **0.67** | **6** | **4.20** | **0.40** |
| **ASB** | **0.88** | **0.89** | **2** | **4.04** | **0.17** | **0.78** | **0.78** | **0** | **4.20** | **0.29** |

Table S2: The calculated values of Schottky barrier height (SBH), electron affinity energy (EAE), and potential step, ∆V, for Au(111)/MoS_2_ contact with a defect-free monolayer (PF) and one containing top (VT) and bottom (VB) single S-vacancies, double S-vacancies (DV), as well as top (ST) and bottom (SB) anti-site defects are reported. The SBH are calculated by the method based on electronic band structure projection (SBH-PJ) and modified Schottky-Mott rule (SBH-HP). The data for the 5x5x4 Au(111)/MoS_2_ samples are calculated by using PBE exchange-correlation functional with and without van der Waals DFT-D2 corrections.

| **Defect type** | **PBE** | | | | | **PBE + van der Waals corrections** | | | | |
| --- | --- | --- | --- | --- | --- | --- | --- | --- | --- | --- |
|  | **SBH-PJ (eV)** | **SBH-HP (eV)** | **Difference (%)** | **EAE (eV)** | **∆V (eV)** | **SBH-PJ (eV)** | **SBH-HP (eV)** | **Difference (%)** | **EAE (eV)** | **∆V (eV)** |
| **PF** | **0.67** | **0.69** | **3** | **4.20** | **0.21** | **0.57** | **0.61** | **7** | **4.36** | **0.30** |
| **VT** | **0.77** | **0.79** | **3** | **4.14** | **0.17** | **0.67** | **0.70** | **4** | **4.31** | **0.26** |
| **VB** | **0.78** | **0.80** | **3** | **4.14** | **0.16** | **0.67** | **0.69** | **3** | **4.31** | **0.27** |
| **DV** | **0.82** | **0.84** | **3** | **4.10** | **0.16** | **0.71** | **0.75** | **6** | **4.25** | **0.27** |
| **AST** | **0.75** | **0.78** | **5** | **4.02** | **0.30** | **0.65** | **0.67** | **3** | **4.20** | **0.40** |
| **ASB** | **0.92** | **0.94** | **3** | **4.00** | **0.16** | **0.82** | **0.84** | **2** | **4.19** | **0.24** |

Table S3: The calculated values of Schottky barrier height (SBH), electron affinity energy (EAE), and potential step, ∆V, for Au(111)/MoS_2_ contact with a defect-free monolayer (PF) and one containing top (VT) and bottom (VB) single S-vacancies, double S-vacancies (DV), as well as top (ST) and bottom (SB) anti-site defects are reported. The SBH are calculated by the method based on electronic band structure projection (SBH-PJ) and modified Schottky-Mott rule (SBH-HP). The data for the 4x4x4 Au(111)/MoS_2_ samples are calculated by using PBE exchange-correlation functional with and without van der Waals DFT-D2 corrections.

| **Defect type** | **PBE** | | | | | **PBE + van der Waals corrections** | | | | |
| --- | --- | --- | --- | --- | --- | --- | --- | --- | --- | --- |
|  | **SBH-PJ (eV)** | **SBH-HP (eV)** | **Difference (%)** | **EAE (eV)** | **∆V (eV)** | **SBH-PJ (eV)** | **SBH-HP (eV)** | **Difference (%)** | **EAE (eV)** | **∆V (eV)** |
| **PF** | **0.67** | **0.69** | **3** | **4.20** | **0.21** | **0.57** | **0.59** | **4** | **4.36** | **0.31** |
| **VT** | **0.81** | **0.83** | **2** | **4.11** | **0.16** | **0.71** | **0.72** | **1** | **4.30** | **0.24** |
| **VB** | **0.82** | **0.83** | **2** | **4.11** | **0.16** | **0.71** | **0.71** | **0** | **4.30** | **0.24** |
| **DV** | **0.87** | **0.88** | **1** | **4.06** | **0.16** | **0.76** | **0.81** | **7** | **4.23** | **0.26** |
| **AST** | **0.77** | **0.81** | **6** | **3.97** | **0.33** | **0.68** | **0.71** | **5** | **4.18** | **0.40** |
| **ASB** | **0.97** | **1.00** | **3** | **3.96** | **0.15** | **0.87** | **0.93** | **6** | **4.18** | **0.21** |

Table S4 The calculated values of Schottky barrier height (SBH), electron affinity energy (EAE), and potential step, ∆V, for Au(111)/MoS_2_ contact with a defect-free monolayer (PF) and one containing top (VT) and bottom (VB) single S-vacancies, double S-vacancies (DV), as well as top (ST) and bottom (SB) anti-site defects are reported. The SBH are calculated by the method based on electronic band structure projection (SBH-PJ) and modified Schottky-Mott rule (SBH-HP). The data for the 3x3x6 Au(111)/MoS_2_ samples are calculated by using PBE exchange-correlation functional with and without van der Waals DFT-D2 corrections.

| **Defect type** | **PBE** | | | | | **PBE + van der Waals corrections** | | | | |
| --- | --- | --- | --- | --- | --- | --- | --- | --- | --- | --- |
|  | **SBH-PJ (eV)** | **SBH-HP (eV)** | **Difference (%)** | **EAE (eV)** | **∆V (eV)** | **SBH-PJ (eV)** | **SBH-HP (eV)** | **Difference (%)** | **EAE (eV)** | **∆V (eV)** |
| **PF** | **0.67** | **0.72** | **7** | **4.18** | **0.20** | **0.57** | **0.61** | **7** | **4.34** | **0.32** |
| **VT** | **0.84** | **0.85** | **1** | **4.10** | **0.15** | **0.75** | **0.78** | **4** | **4.28** | **0.21** |
| **VB** | **0.85** | **0.86** | **1** | **4.09** | **0.15** | **0.75** | **0.78** | **4** | **4.28** | **0.21** |
| **DV** | **0.90** | **0.92** | **3** | **4.04** | **0.14** | **0.80** | **0.83** | **4** | **4.20** | **0.24** |
| **AST** | **0.82** | **0.85** | **4** | **3.92** | **0.33** | **0.71** | **0.73** | **2** | **4.16** | **0.38** |
| **ASB** | **1.00** | **1.01** | **1** | **3.92** | **0.14** | **0.91** | **0.93** | **3** | **4.16** | **0.18** |

# Effect of defect density on SBH

We plot the pDOS for Mo- and S-atoms of an MoS_2_ monolayer with bottom single S-vacancies, double S-vacancies, and bottom antisite Mo_S_ defects at various defect densities (per unit area of MoS_2_ ) in Figure S1, Figure S2 and Figure S3, respectively. As can be seen in Figure S1, the overall shape of pDOS for the MoS2 monolayer with bottom S-monovacancies varies with the defect density: the height of peak within the band gap increases proportionally with the defect density. The similar changes in the pDOS Au(111)/MoS_2_ monolayer with S-divacancies with defect density are shown in Figure S2: the height of peaks in the band gap increases. An additional peak close to the bottom of conduction band appears at the highest vacancy density.


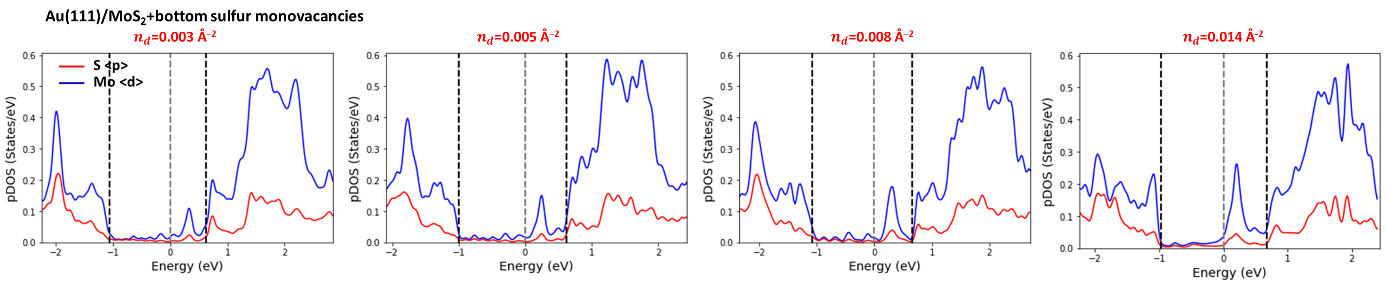


Figure S1: PDOS of Au(111) /MoS2 sample with a monolayer containing bottom single sulfur vacancies at different defect densities. The pDOS calculated as an average over five d-orbitals of Mo-atoms indicated by blue, and over three p-orbitals of S-atoms indicated by red. The VBM, Fermi level and CBM, obtained with the PJ method, are shown by dashed lines. The PDOS are for DFT calculations with DT2 van der Waals corrections.


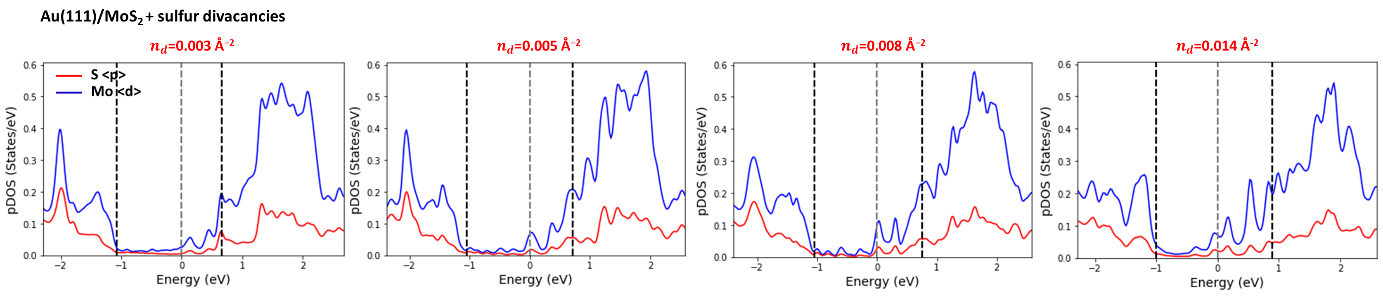


Figure S2: PDOS of Au(111)/MoS2 sample with a monolayer containing bottom double sulfur vacancies at different defect densities. The pDOS calculated as an average over five d-orbitals of Mo-atoms indicated by blue, and over three p-orbitals of S-atoms indicated by red. The VBM, Fermi level and CBM, obtained with the PJ method, are shown by dashed lines. The PDOS are for DFT calculations with DT2 van der Waals corrections.

The pDOS for of Mo- and S- atoms in the Au(111)/MoS_2_ sample with a monolayer containing bottom anti-site defects at different defect densities are shown in Figure S3. The interaction of the anti-site defects with the underlying gold substrate is stronger as compared with the other studied defects. As a result, many states appear in the band gap, forming the broad continuous-like spectrum of states as shown in Figure S3. The density of the introduced states increases in direct proportion with defect concentration. However, the CBM position is clearly visible in the pDOS, and it can be used to demonstrate the increase in SBH with defect concentration.


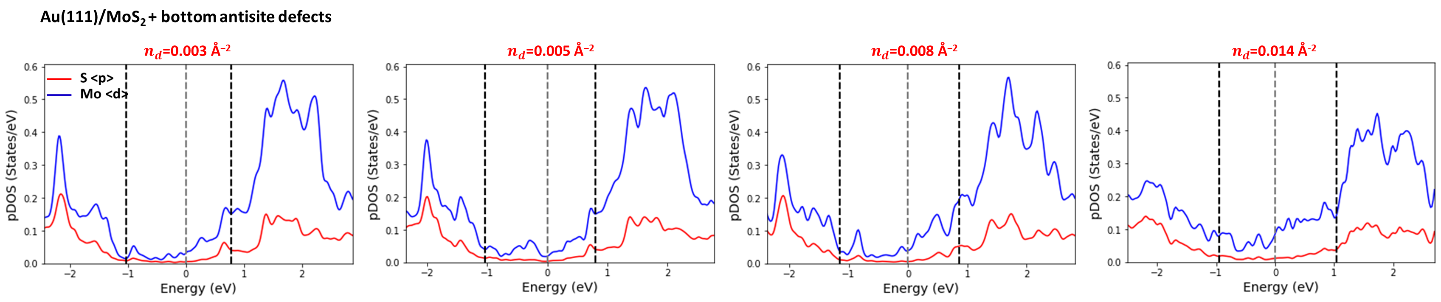


Figure S3: pDOS of Au(111) /MoS_2_ sample with a monolayer containing bottom anti-site defects at different defect densities. The PDOS calculated as an average over five d-orbitals of Mo-atoms indicated by blue, and over three p-orbitals of S-atoms indicated by red. The VBM, Fermi level and CBM, obtained with the PJ method, are shown by dashed lines.

The effect of defect density per unit area on the SBH obtained by DFT calculations with PBE XC-functional by using the method based on projection of electronic band structure is reported in Figure S4 (a). The SBH values for Au/MoS_2_ sample with a MoS_2_ monolayer containing single top (blue circles) and bottom (magenta circles) sulfur vacancies, double sulfur vacancies (green triangles), as well as top (black squares) and bottom (red squares) anti-site Mo_S_ defects are shown in Figure S4 (b). The SBH value of the Au(111)/MoS_2_  heterojunction with a defect-free MoS_2_ is given for comparison.

# The effect of different point defects on the SBH

The effect of point defects on SBH is illustrated in Figure S4 (a). The SBH value for Au(111)/MoS_2_ heterojunction with a defect-free MoS_2_ monolayer and a monolayer containing single top and bottom sulfur monovacancies, sulfur divacancies, as well as top and bottom anti-site Mo_S_ defects are shown in Figure S4 (b). The DFT calculations with PBE XC-functional are reported obtained by the PJ method based on projection of electronic band structure.


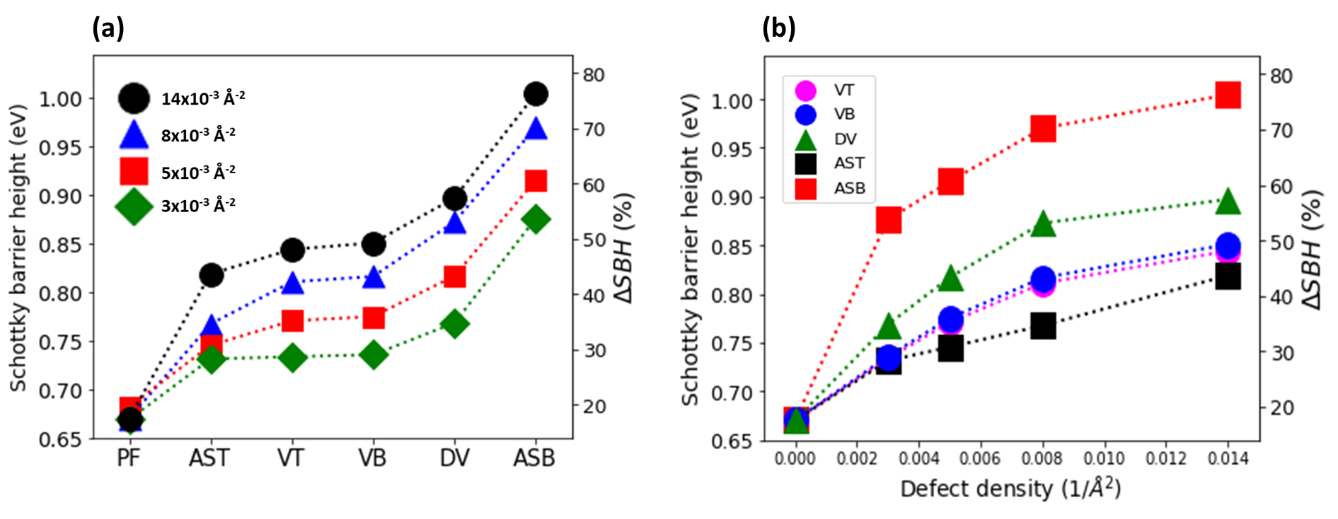


Figure S4: (a) The effect of concentration of point defects on the SBH. The SBH value for Au(111) /MoS_2_ contact with a defect-free monolayer (PF) and a monolayer containing single top (VT) and bottom (VB) sulfur vacancies, double sulfur vacancies (DV), as well as top (AST) and bottom (ASB) anti-site defects. The data is from 3x3 samples with gold 6 layers (black circles), and 5x5 (red squares), 4x4 (blue triangles), and 6x6 (green diamonds) Au/MoS_2_ samples with gold 4 layers. (b) The effect of defect concentration on the SBH for the Au(111)/MoS_2_ samples with a MoS_2_ monolayer containing single top (blue circles) and bottom (clue circles) sulfur vacancies, double sulfur vacancies (green triangles), as well as top (black squares) and bottom (red squares) anti-site defects. The SBH value of the defect-free sample is given for comparison. The reported SBH values were obtained by DFT calculations with PBE XC-functional using the method based on projection of electronic band structure.

Table S5: Increase in the value of SBH in defective MoS_2_ monolayer. The reported data for DFT calculations with PBE and PBE with van der Waals corrections. The electronic band structure projection method is used.

| **Defect type** | **∆ SBH (%) {PBE}** | | | | **∆ SBH (%) {PBE + van der Waals corrections}** | | | |
| --- | --- | --- | --- | --- | --- | --- | --- | --- |
|  | $\boldsymbol{n}_{\boldsymbol{d}}$**=0.003 (1/Å^2^)** | $\boldsymbol{n}_{\boldsymbol{d}}$**=0.005 (1/Å^2^)** | $\boldsymbol{n}_{\boldsymbol{d}}$**=0.008 (1/Å^2^)** | $\boldsymbol{n}_{\boldsymbol{d}}$**=0.014 (1/Å^2^)** | $\boldsymbol{n}_{\boldsymbol{d}}$**=0.003 (1/Å^2^)** | $\boldsymbol{n}_{\boldsymbol{d}}$**=0.005 (1/Å^2^)** | $\boldsymbol{n}_{\boldsymbol{d}}$**=0.008 (1/Å^2^)** | $\boldsymbol{n}_{\boldsymbol{d}}$**=0.014 (1/Å^2^)** |
| **VT** | **9%** | **13%** | **21%** | **26%** | **13%** | **18%** | **25%** | **32%** |
| **VB** | **10%** | **14%** | **22%** | **27%** | **13%** | **17%** | **25%** | **32%** |
| **DV** | **15%** | **20%** | **30%** | **34%** | **18%** | **24%** | **33%** | **40%** |
| **AST** | **9%** | **10%** | **15%** | **22%** | **11%** | **14%** | **19%** | **25%** |
| **ASB** | **31%** | **35%** | **45%** | **50%** | **37%** | **44%** | **53%** | **59%** |

# **Comparison of the two calculation methods and the effect of van der Waals corrections**

Figure S5 (a-d) compare the SBH values calculated with the method based on projection of electronic band structure of MoS_2_ monolayer on band structure of Au(111)/MoS2 contact (blue circles) and with the method based on Hartree potential and the modified Schottky-Mott rule (red squares). In Table S5, we compare the SBH values calculated with the PBE exchange-correlation (XC) potential and PBE-XC with van der Waals DFT-D2 corrections obtained by the method based on projection of electronic band structure.

#
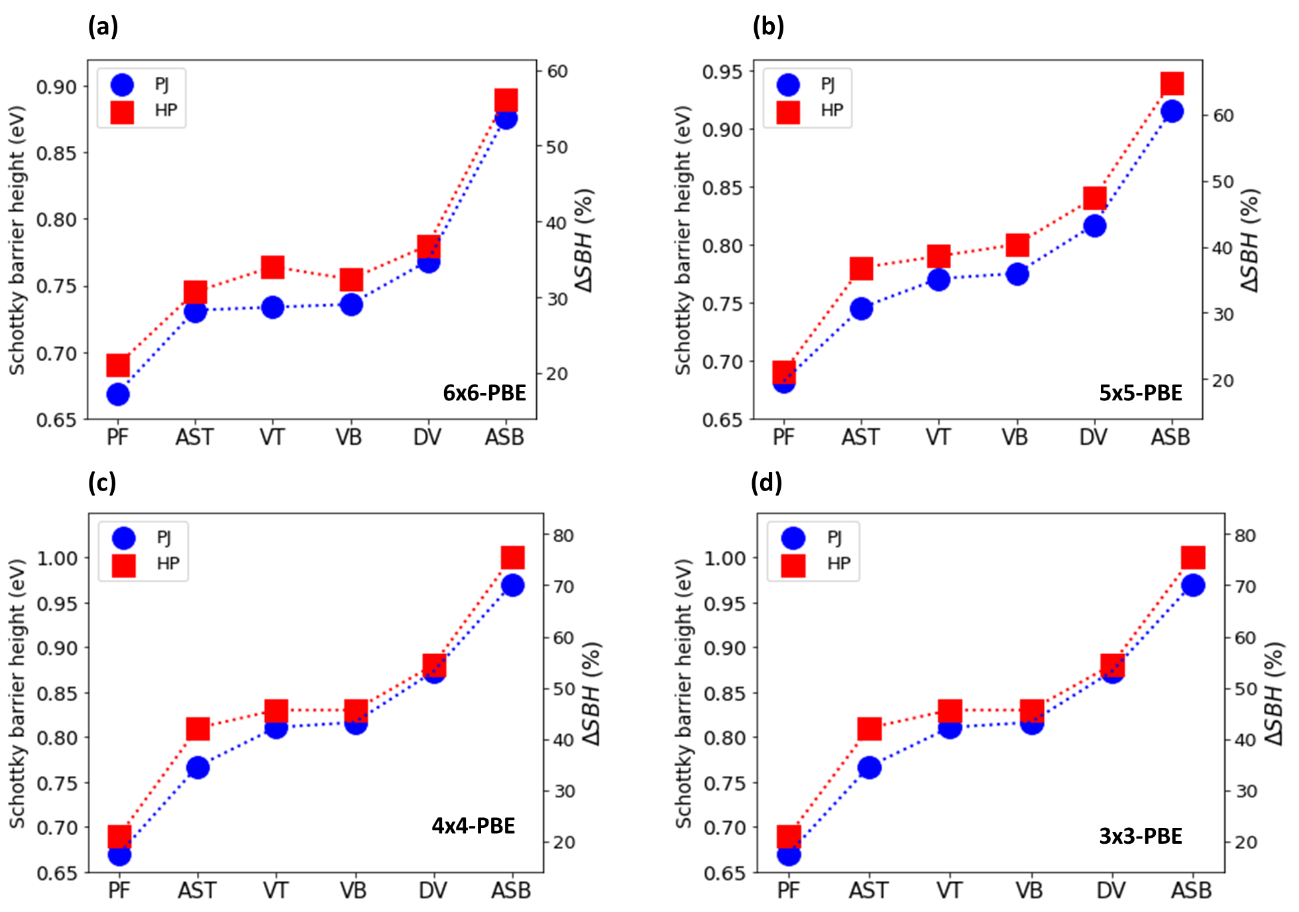


Figure S5: Comparison of the SBH values calculated with the method based on projection (PJ) of electronic band structure of MoS2 monolayer on band structure of Au/MoS2 contact (blue circles) and with the method based on Hartree potential (HP) and the modified Schottky-Mott rule (red squares) for (a) 6x6x4, (b) 5x5x4, (c) 4x4x4 and (d) 3x3x6 Au(111)/MoS_2_ sample with PBE XC-functional.


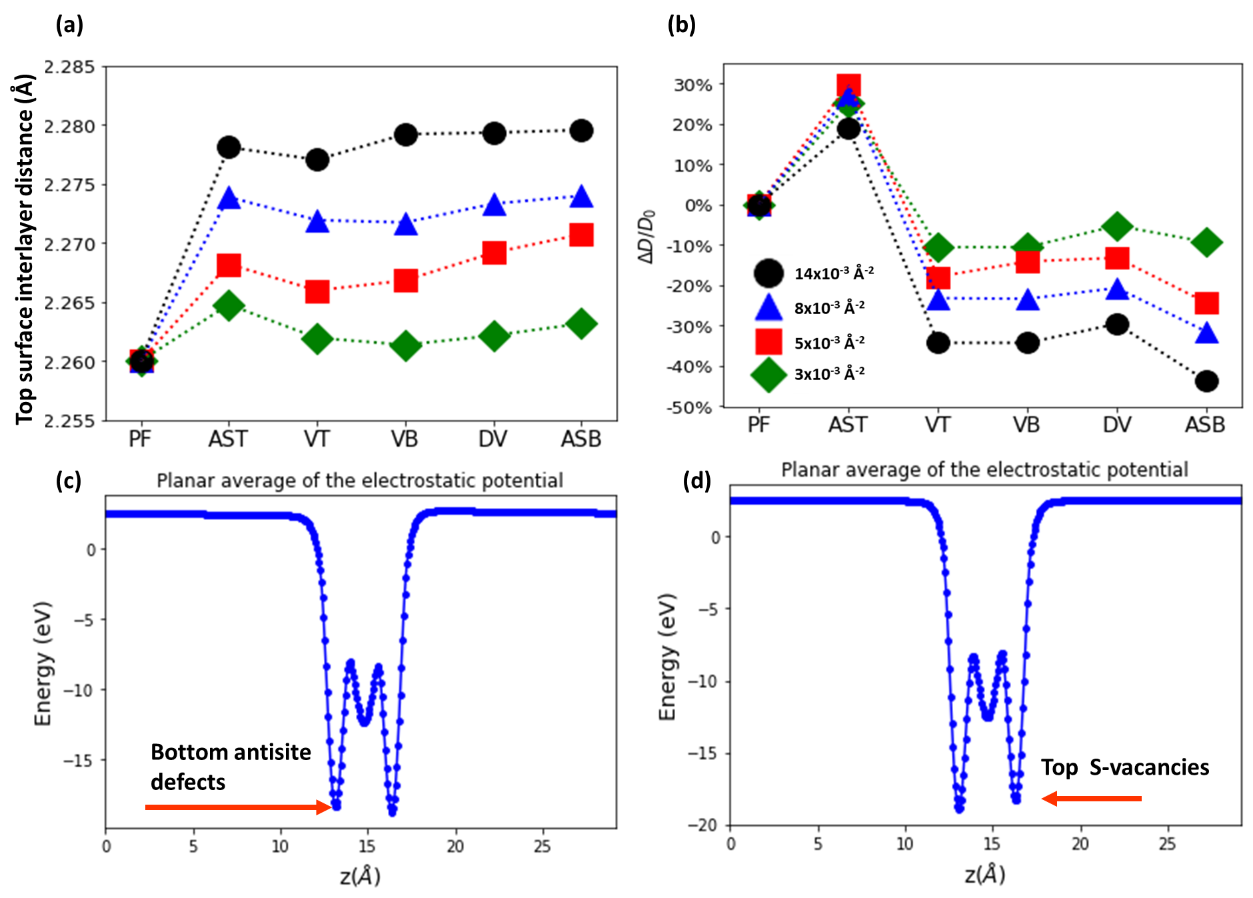


Figure S6: (a) Interlayer distance between the two top adjacent layers of Au(111) surface in contact with defect-free (PF) and defective MoS_2_ monolayer. Defective monolayer contains single top (VT) and bottom (VB) sulfur vacancies, double sulfur vacancies (DV), as well as top (AST) and bottom (ASB) antisite defects. The defect densities are indicated in the inset (b). Variation of the interfacial dipole moment of the defective MoS_2_ layer with respect to the value of defect-free monolayer: $\frac{\boldsymbol{\Delta D}}{\boldsymbol{D}_{\boldsymbol{0}}}\boldsymbol{=}\mathbf{100}\boldsymbol{*}\left( \frac{\boldsymbol{D-}\boldsymbol{D}_{\boldsymbol{0}}}{\boldsymbol{D}_{\boldsymbol{0}}} \right)$ (c, d) Planar average of Hartree potential for Au(111)/MoS_2_ sample with the MoS_2_ monolayer containing a bottom antisite defect (c) and a single top vacancy (d). The Z-axis is normal to the Au(111)/MoS2 interface; the plane average is calculated over [XY] planes along the sample. The plots are for the Au(111)/MoS2 6x6x4 samples.


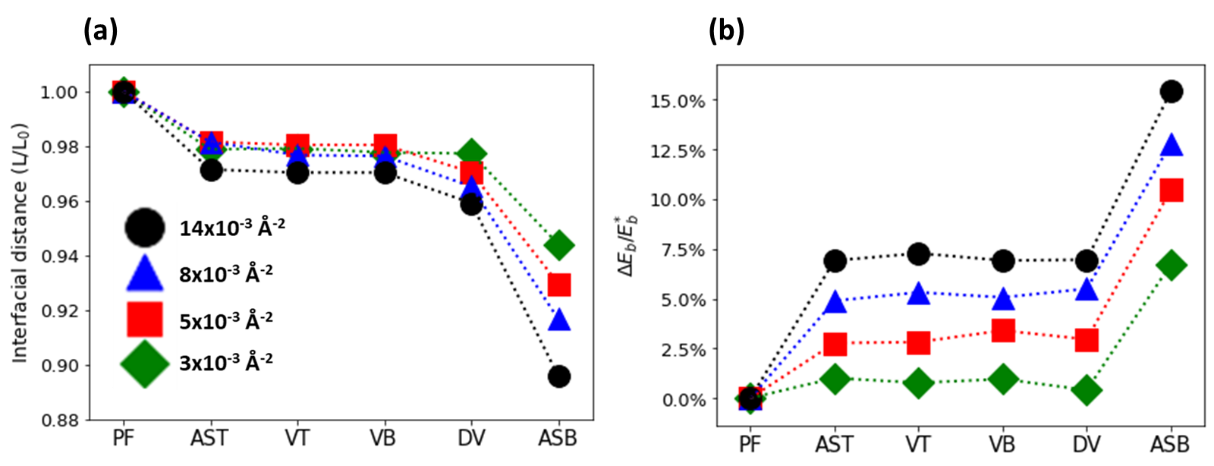


Figure S7: (a) Interfacial distance for the Au(111)/MoS_2_ sample with defect-free (PF) and defective MoS_2_ monolayer. Defective monolayer contains single top (VT) and bottom (VB) sulfur vacancies, double sulfur vacancies (DV), as well as top (AST) and bottom (ASB) antisite defects. The defect densities are indicated in the inset. (b) Variation of the binding energy, $\boldsymbol{E}_{\boldsymbol{b}}$, of the defective MoS_2_ layer with respect to the value of defect-free monolayer, $\boldsymbol{E}_{\boldsymbol{b}}^{\boldsymbol{*}}$: $\frac{\boldsymbol{\Delta E}_{\boldsymbol{b}}}{\boldsymbol{E}_{\boldsymbol{b}}^{\boldsymbol{*}}}\boldsymbol{=}\mathbf{100}\boldsymbol{*}\left( \frac{\boldsymbol{E}_{\boldsymbol{b}}\boldsymbol{-}\boldsymbol{E}_{\boldsymbol{b}}^{\boldsymbol{8}}}{\boldsymbol{E}_{\boldsymbol{b}}^{\boldsymbol{*}}} \right)$ .

1. Email: sorkinv@ihpc.a-star.edu.sg [↑](#footnote-ref-1)
2. Email: kahwee.ang@nus.edu.sg [↑](#footnote-ref-2)
3. Email: zhangyw@ihpc.a-star.edu.sg [↑](#footnote-ref-3)
